# Boronic Acid-Containing 3*H*- pyrazolo[4,3-*f*]quinoline Compounds as Dual CLK/ROCK Inhibitors with Anticancer Properties

**DOI:** 10.3390/ph17121660

**Published:** 2024-12-10

**Authors:** Neetu Dayal, Riddhi Chaudhuri, Kofi Simpa Yeboah, Nickolas R. Brauer, Herman O. Sintim

**Affiliations:** 1 Department of Chemistry, Purdue University, 560 Oval Drive, West Lafayette, IN 47907, USA; nitiapi@gmail.com (N.D.); chaudhu5@purdue.edu (R.C.); yeboahk@purdue.edu (K.S.Y.); brauer4@purdue.edu (N.R.B.); 2 Purdue Institute for Drug Discovery, Purdue University, 720 Clinic Drive, West Lafayette, IN 47907, USA; 3 Purdue Institute for Cancer Research, Purdue University, 201 S. University Street, West Lafayette, IN 47907, USA

**Keywords:** boron, Povarov/Doebner multicomponent reaction, kinase inhibitor, oncogenic kinase, CLK, ROCK, DNA damage, cell cycle arrest, anticancer

## Abstract

**Background:** The protein kinases CLK and ROCK play key roles in cell growth and migration, respectively, and are potential anticancer targets. ROCK inhibitors have been approved by the FDA for various diseases and CLK inhibitors are currently being trialed in the clinic as anticancer agents. Compounds with polypharmacology are desired, especially in oncology, due to the potential for high efficacy as well as addressing resistance issues. In this report, we have identified and characterized novel, boron-containing dual CLK/ROCK inhibitors with promising anticancer properties. **Methods:** A library of boronic acid-based CLK/ROCKi was synthesized via Povarov/Doebner-type multicomponent reactions. Kinase inhibition screening and cancer cell viability assays were performed to identify the hit compounds. To gain insights into the probable binding modes of the compounds to the kinases, docking studies were performed. Cell cycle analysis, qPCR and immunoblotting were carried out to further characterize the mode(s) of action of the lead candidates. **Results:** At 25 nM, the top compounds **HSD1400** and **HSD1791** inhibited CLK1 and 2 and ROCK2 at greater than 70%. While **HSD1400** also inhibited CLK4, the C1 methylated analog **HSD1791** did not inhibit CLK4. Antitumor effects of the top compounds were evaluated and dose–response analysis indicated potent inhibition of renal cancer and leukemia cell growth. Immunoblotting results indicated that the top compounds induce DNA damage via upregulation of p-H2AX. Moreover, flow cytometry results demonstrated that the top compounds promote cell cycle arrest in the renal cancer cell line, Caki-1. qPCR and immunoblotting analysis upon **HSD1791** dosing indicated suppression of cyclin D/Rb oncogenic pathway upon compound treatment. **Conclusions:** Novel boronic acid-containing pyrazolo[4,3-*f*]quinoline-based dual CLK/ROCK inhibitors were identified. The so-called “magic methylation” design approach was used to tune CLK selectivity. Additionally, the findings demonstrate potent in vitro anticancer activity of the lead candidates against renal cancer and leukemia. This adds to the growing list of boron-containing compounds that display biological activities.

## 1. Introduction

**Figure 1:**
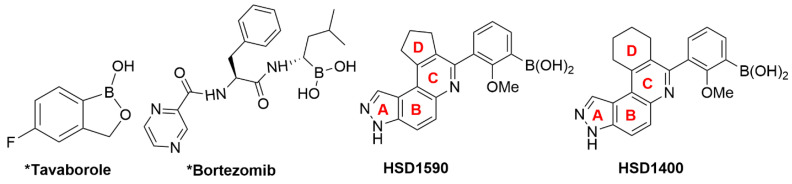
Structures of boronic acid-containing compounds, reported in the literature (e.g., bortezomib, **HSD1590**) and characterized in this study (**HSD1400**). * indicates FDA-approved.

Boronic acid or ester moieties are versatile functional groups, which are used in various transformations in organic chemistry [1,2,3,4,5,6]. The boronic acid or ester groups have also emerged as important units in advanced materials [2]. Some FDA-approved drugs, such as bortezomib, ixazomib (used in refractory multiple myeloma treatment), crisaborole (used in atopic dermatitis treatment), vaborbactum (used in combination with other antibiotics) and tavaborole (fungal infection treatment), contain boron and this has further motivated the exploration of boron-containing chemotypes in medicine (selected structures in Figure 1) [7,8,9]. Boron compounds act as privileged scaffolds and diverse subclasses have been explored in medicinal chemistry, including boronic acids (BA), boronic esters and oxaboroles [8]. Recently, boron functional groups have been incorporated in prodrugs and carrier moieties (such as liposomal, vesicular or nanocarriers) to improve bioavailability, which further broadens the therapeutic applications of boron [9]. It is important to note that the chemical nature of the B atom governs its biological activity since BAs have empty p orbitals in their sp^2^-hybridized state; under physiological conditions, they can interconvert to sp^3^ states (tetragonal geometry) via interaction with nucleophiles resulting in covalent interactions, for instance, with OH groups of Ser and Thr and also SH group of Cys residues at the enzyme active sites. Interestingly, these covalent interactions are reversible [10]. Moreover, OH groups in BA afford multiple hydrogen bonding interactions thereby promoting target affinity and reducing the probability of the development of drug resistance in case of receptor mutations [10]. Since BAs can adopt diverse modes of bonding interactions with their targets in biological environments, they are referred to as a “magic element” in drug design; further exploration of novel BA-containing scaffolds could lead to interesting drug candidates [10].

**Figure 2:**
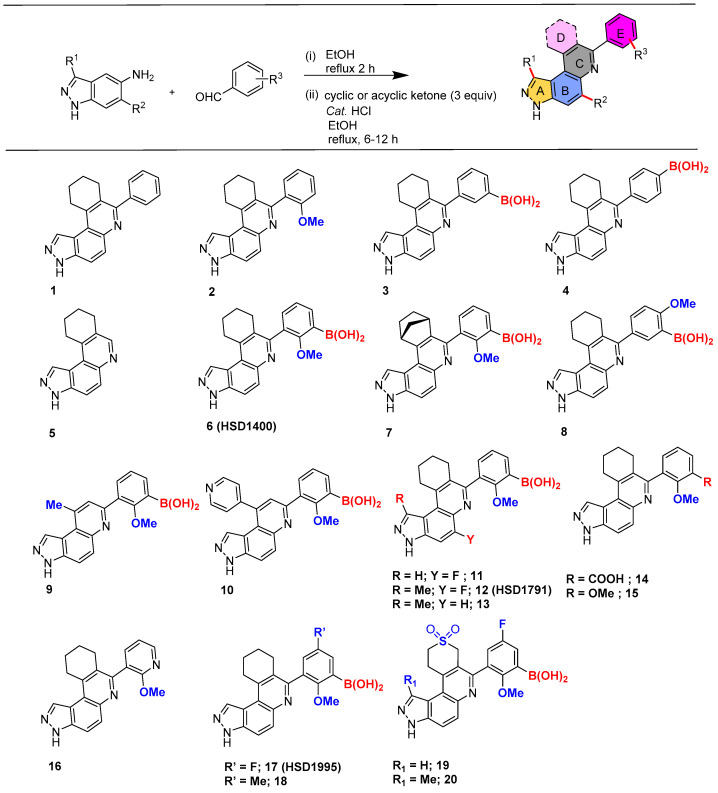
Structures of **HSD1400** analogs.

**Figure 3:**
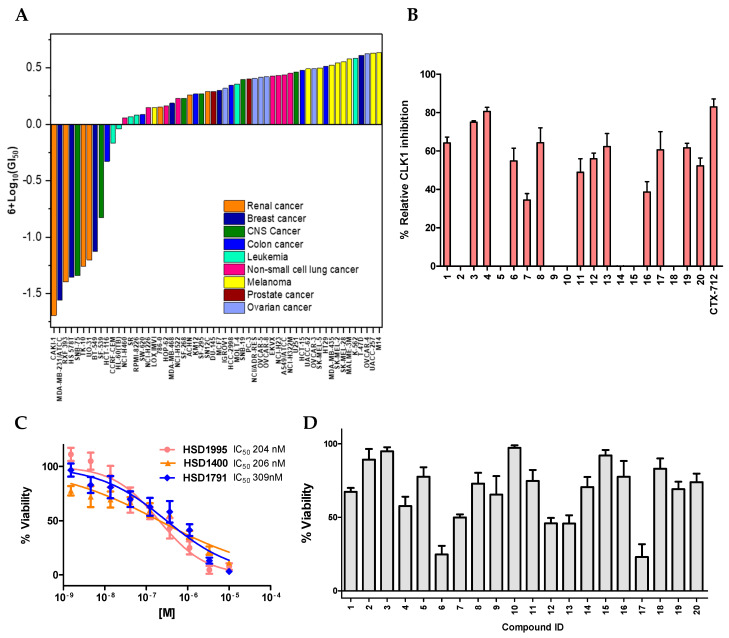
(**A**) NCI-60 tumor cell line screen of **HSD1400**. (**B**) ADP-Glo assay against CLK1 at 200 nM compound concentration; error bars represent mean ± SD. (**C**) Dose–response of **HSD1400** analogs against Caki-1 treated with the respective compounds for 72 h; error bars represented by mean ± SD. (**D**) Caki-1 viability upon 1 μM compound treatment for 72 h; experiment was performed in triplicates, and error bars indicate mean ± SD.

**Figure 4:**
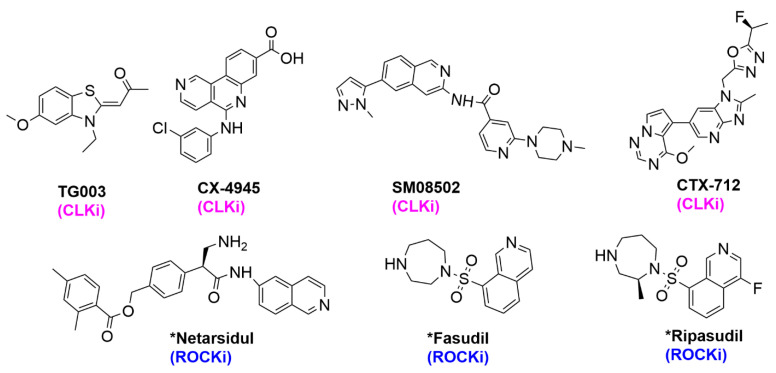
Structures of CLK/ROCKi inhibitors reported in the literature, * indicates FDA approval.

We have previously developed a simple, fast approach to make a library of BA-containing 3*H*- pyrazolo[4,3-*f*]quinoline compounds via Povarov/Doebner-type reactions [11]. This type of multi-component reaction (MCR) strategy allows one to quickly make potential drug candidates using readily available starting materials: amines, aldehydes and ketones [11]. In the Povarov MCR, amines and aldehydes react to yield Schiff bases, which in turn react with cyclic or acyclic ketones to produce the quinoline-containing final products (reaction scheme in Figure 2). Using this strategy, we previously identified **HSD1590** (structure in Figure 1) as an ultrapotent ROCK inhibitor [11]. Changing the D ring in **HSD1590** from a five-membered to a six-membered ring afforded **HSD1400**, which is also a ROCKi with superior anticancer activities when profiled against the NCI-60 panel (see Figure 3A). **HSD1400** was highly active against renal cancer cell lines, such as Caki-1 with IC_50_ of 206 nM (Figure 3C). Further kinome screening revealed that in addition to ROCK, the 3*H*- pyrazolo[4,3-*f*]quinoline-containing compounds also inhibit CLKs (SI). Thus, **HSD1590**, **HSD1400** and close analogs are dual CLK/ROCK inhibitors. ROCKs are emerging as important anticancer targets as they regulate diverse pathways in tumorigenesis including cell migration and metastasis [12,13]. While a few FDA-approved ROCKi are available (structures in Figure 4), the potencies of some, such as fasudil, are weak and/or their kinome selectivity is not great; hence, the development of additional ROCK inhibitors would be welcome [13]. CLK kinases have also emerged as potential anticancer targets, and while no CLKi has been approved yet by the FDA, a few CLK inhibitors (see Figure 4) have entered clinical trials (such as NCT03355066) [14,15,16,17]. We were particularly excited about the prospect of the boronic acid-containing pyrazolo[4,3-*f*]quinoline compounds inhibiting CLKs because early clinical data indicate that CLK inhibitors, such as CTX-712, have an acceptable safety profile, and crucially, signs of clinical antitumor activities have been observed [16]. Therefore, we initiated a more expansive campaign to evaluate how various modifications to the pyrazolo[4,3-*f*]quinoline core affected CLK inhibition (Figure 2 and Figure 3B) and the cell viability of selected cancer cell lines.

To evaluate the antiproliferative effects of the hit CLK/ROCKi, NCI-60 tumor screening was performed which revealed the potency of the top compounds against difficult-to-treat cancers, including renal cancer [18]. This is especially relevant since preliminary studies have shown CLK1 mRNA levels are upregulated in renal tumors resulting in poor disease prognosis, moreover, increased expression of SR proteins (downstream targets of CLKs) such as SRSF1 is associated with renal carcinogenesis [19,20]. However, the antitumor effects of CLK inhibition have not yet been thoroughly evaluated against RCC. This study provides mechanistic insight into the antitumor activity of the lead CLK/ROCKi via docking and in vitro target engagement studies. Additionally, the induction of cell cycle arrest via suppression of the cyclin D/Rb pathway was observed upon treatment of the top CLK/ROCKi, thereby providing deeper mechanistic insight into the tumor cell growth inhibition effects of the lead candidates.

## 2. Results and Discussion

### 2.1. Structure–Activity Relationship (SAR) Study of the In-House Boronic Acid Compounds with Respect to Kinase Inhibition and Growth Suppression in Caki-1

We proceeded to perform an extensive SAR analysis of the first-generation CLK/ROCKi. ADP-Glo assay was performed to delve into the SAR by evaluating the **HSD1400** analogs (Figure 2) against CLK1 at 200 nM (Figure 3B), 100 nM and 50 nM concentrations (Supporting Information, Figure S1B,C). Deleting the 3-borono-2-methoxyphenyl group (i.e., ring E, see Figure 2) in **HSD1400** to afford compound **5** abrogated CLK1 inhibition (Figure 3B). Compound **1**, which contained an unsubstituted phenyl group (i.e., without the methoxy or borono groups) displayed similar CLK1 inhibition (Figure 3B). However, compound **1** was not good at killing cancer cells (*vide infra*, Figure 3D), probably due to poor cell permeation and/or instability in cells. Substituting compound **1** with the methoxy group at the ortho position (compound **2**) killed CLK1 activity but the boron-substituted analogs at the meta and para positions (compounds **3** and **4**) were potent CLK1 inhibitors (Figure 3A). Interestingly the pyridyl analog of compound **2** (compound **16**) had CLK1 inhibition activity, revealing that the deleterious effects of the methoxy group could be context-dependent. Also, having both methoxy and borono substituents (**HSD1400** and compound **8**) maintained CLK1 inhibition so it appears that while the methoxy group was a negative factor for CLK inhibition in compound **2**, placing a BA group next to the methoxy moiety eliminated the deleterious effects of the methoxy group. This is not surprising as functional groups can interact in a synergistic manner; in this case, the methoxy group could donate electrons into the empty *p*-orbital on the boron to give a moiety that has different characteristics from the individual groups. As Woodward would put it, “enforced propinquity could lead to greater intimacy” [21]. This synergy hypothesis is given credence by the fact that when the BA moiety was changed to other groups such as carboxylic acid or methoxy (compounds **14** and **15**), CLK1 inhibition was abrogated (Figure 3B). Fluorine and methyl groups are so-called “magic” in drug design and so we made analogs **17** and **18** with fluorine substitution (**17**) and methyl substitution (**18**) of the 3-borono-2-methoxyphenyl group. Compound **17** was active against CLK1 while **18** was not. Another “methyl” or “fluorine” scan on ring A (methyl scan) and ring B (fluorine scan) afforded compounds **11**, **12** (also called **HSD1791**) and **13**, which were all active against CLK1.

The cyclohexyl moiety (ring D in **HSD1400**) appears critical for CLK inhibition since we observed reduced potency (compound **7**) or complete loss in activity (compounds **9** and **10**) upon substitution with a bicyclic group (compound **7**), methyl (**9**) or pyridyl (**10**) groups. Many drugs with sulfonyl moiety have been reported and this group lowers the lipophilicity of compounds (lower logP) and/or could improve the aqueous solubility of compounds. Therefore, we made analogs **19** and **20** whereby the six-membered ring D contained a sulfonyl moiety. Compounds **19** and **20** could also inhibit CLK1 (Figure 3B), but unfortunately, the sulfonyl analogs **19** and **20** were not active against the Caki-1 cell line.

We had earlier sent **HSD1400** out for NCI-60 screening and this revealed that **HSD1400** (compound **6**), is active against renal cell lines, such as Caki-1 (Figure 3A). This contrasted with the poor inhibition of Caki-1 by an earlier reported CLK inhibitor TG003, which required very high concentrations (>30 μM) to achieve ~60% cell growth reduction (Supplementary Information (SI, Figure S1D)). In general, compounds that displayed poor CLK1 inhibition (compounds **2**, **5**, **9**, **10**, **14**, **15**, **18**) also displayed poor inhibition of Caki-1 (Figure 3D). Growth inhibition studies against Caki-1 revealed ring D needs to be a cyclohexyl group as substitutions with methyl and pyridyl moieties are not tolerated (compounds **9** and **10**), and as previously stated heteroatom substitution of ring D to sulfonyl moieties, resulted in poor Caki-1 viability (compounds **19** and **20**).

Given the potency of the CLK inhibitors against Caki-1, we also extended the study to 786-O and ACHN renal carcinoma cell lines and assessed viability against the top CLK inhibitors, compounds **3**, **4**, **6**, **8**, **12**, **13**, **17**, **19** (SI, Table S1). Overall, we found both the cell lines were sensitive to the CLK inhibitors with ACHN exhibiting greater sensitivity against the compounds. **HSD1400** exhibited the highest potency; at a 1 µM dose, the percent of inhibition was greater than 40%, increasing to greater than 80% at 10 µM against ACHN and 786-O, respectively (SI, Table S1). Furthermore, **HSD1995** and HSD**1791** (compounds **17** and **12**, respectively) were among the top hits across all three cell lines (Caki-1, ACHN and 786-O). **HSD1400**, **HSD1995** and **HSD1791** were selected as the top compounds for further characterization. Given the remarkable sensitivity of Caki-1 (submicromolar activity) towards the top compounds, we evaluated the dose response in the Caki-1 cell line (IC_50_ ranging from 204 to 309 nM) (Figure 3C).

### 2.2. Novel Pyrazolo Quinoline-Based Compounds **HSD1400** and **HSD1791** Are Dual CLK/ROCKi

**Figure 5:**
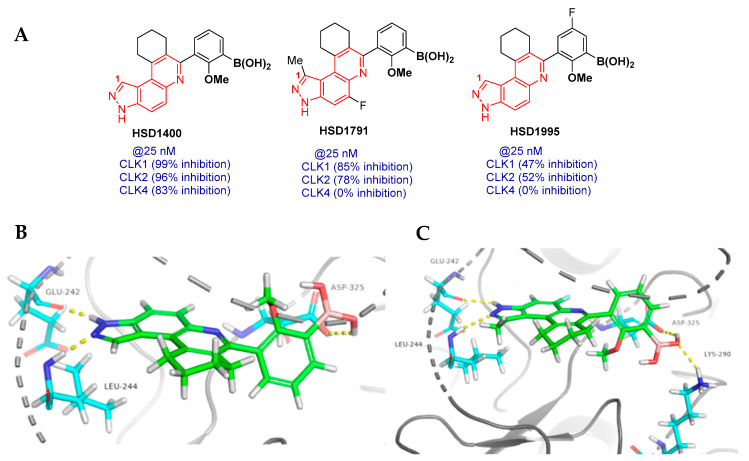
(**A**) Structures of hit pyrazolo-quinoline-based CLK inhibitors. Kinase assay to determine CLK inhibition was performed by Reaction Biology. (**B**) **HSD1400** docked to CLK1. (**C**) **HSD1791** docked to CLK1.

The preliminary kinase assay of the first-generation BA-containing compounds revealed **HSD1400**, **HSD1995** and **HSD1791** were among the top compounds identified with activity against CLK1 (Figure 3A and Figure S1). To confirm the results, a more sensitive kinase assay was employed by Reaction Biology that revealed that **HSD1400** and **HSD1791** potently inhibit CLK activity, along with ultrapotent ROCK inhibition (Figure 5A, SI, Table S2). We have earlier revealed that substitution of the C1 position of the 3*H*-pyrazolo[4,3-*f*]quinoline moiety afforded a more selective kinase inhibitor [22]. Indeed, kinase profiling of the first-generation BA-containing compounds revealed that substituting the C1 position with methyl yielded **HSD1791**, which is a more selective CLK inhibitor (Figure 5A, SI, Tables S2–S4).

To obtain insights into how the compounds may bind to CLK, molecular docking analysis of the selected compounds bound to CLK1 and 4 was performed (Figure 5B,C, Supplementary Information Figures S2 and S3 and Table S5). While we are aware that one must be cautious in relying overly on docking scores, we note that the docking score results aligned with the kinase inhibition data as **HSD1400** and **HSD1791** were among the top-scoring compounds (Table S5). Furthermore, we delved into the possible interaction of the top compounds with the active site of CLK1/4. CLK1 and CLK4 have been shown to be highly homologous in amino acid sequence and tertiary structure [23]. Therefore, docking analyses were performed to further investigate the source of selective inhibition of CLK1 over CLK4 by **HSD1791** in comparison to **HSD1400**. Compounds expressing the pyrazolo[4,3-*f*]quinoline moiety have been shown to bind to the active conformation of CDK2/Cyclin A2 [22]; therefore, crystal structures exhibiting the active conformation of CLK1 (PDB: 6QTY) and CLK4 (PDB: 6FYV) were chosen to perform molecular docking. Both **HSD1400** and **HSD1791** generated strong hydrogen bonding interactions to hinge residues Glu 242 and Leu 244 using the pyrazole moiety in both CLK1 (Figure 5B,C) and CLK4 (SI, Figure S2). Additionally, the BA moiety in **HSD1400** and **HSD1791** form hydrogen bonds with DFG residue Asp 325. In CLK1, **HSD1791** participates in an extra hydrogen bonding interaction with Lys 290 which can be attributed to the orientation of the BA moiety directed toward the C-lobe (Figure 5C), which deviates from the **HSD1400** binding conformation (Figure 5B). The additional interaction observed in **HSD179**1 does not translate into biochemical activity; however, in CLK4, the BA group of **HSD1400** participates in hydrogen bonding interactions with Asp 325 and Ala 171 (SI, Figure S2). **HSD1791** also forms a hydrogen bond with Asp 325; however, a few clashing interactions form (SI, Figure S2), which might explain the reduced potency against CLK4 (Figure 5A). We are cautious that the 3-borono-2-methoxyphenyl group in the docked pose of **HSD1400** and **1791** is oriented differently and experimental verification of the bound complexes, beyond the scope of this manuscript, is needed to explain the CLK selectivities of the compounds.

### 2.3. **HSD1400** Analogs Promote DNA Damage in Caki-1 Cells

**Figure 6:**
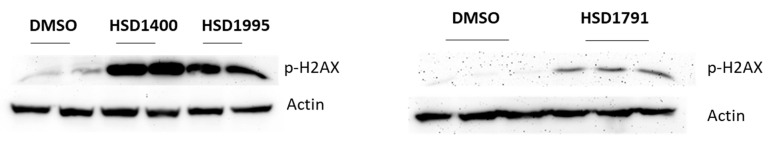
Western blotting analysis in Caki-1, cells were treated with 5 μM compounds for 24 h, assay included 2–3 biological replicates per experimental group.

A previous study has shown that alternative splicing levels of specific DNA damage response genes are altered upon CLK1 inhibition [20]. Furthermore, phosphorylation of Aurora B by CLKs 1, 2 or 4 plays a key role in preventing DNA damage during cell division. CLK inhibition has been shown to accelerate midbody disassembly and chromatin breakage thereby inducing DNA damage in segregating cells [24]. Therefore, we were motivated to understand if the lead compounds induce DNA damage in Caki-1. Mechanistic insight by Western assays revealed that DNA damage induction occurs via increased p-H2AX levels (Figure 6). The CLK inhibitor, cirtuvivint, currently in clinical trials also induced DNA damage in Caki-1 via p-H2AX upregulation (SI, Figure S4) [25].

### 2.4. **HSD1400** and **HSD1791** Induce Cell Cycle Arrest and Have Additional Antiproliferative Activity Against Leukemia Cell Lines

**Figure 7:**
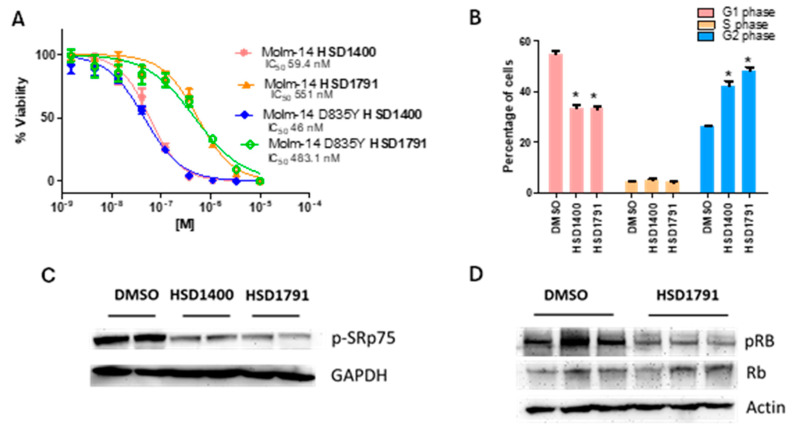
(**A**) Dose–response analysis of the lead candidates against leukemia cell lines at 72 h; error bars represent mean ± SD. (**B**) Caki-1 was treated with 5 μM compounds for 24 h and harvested for flow cytometry; assay included 3 replicates, and error bars represent mean ± SD. * indicates *p* < 0.05. (**C**) Western analysis in Caki-1 at 6 h compound treatment (5 μM). (**D**) Representative western analysis in Caki-1 upon treatment of compounds at 24 h; assay performed with two or more independent with biological replicates per experimental group. Full blot is shown in SI, Figure S6.

Given the potent CLK inhibition as well as renal cancer cell line growth inhibition by **HSD1400/1791**, they were selected for additional experiments. Previous studies have demonstrated that small molecule CLK inhibitors like TG003 and cirtuvivint promote the dephosphorylation of SR proteins [26,27]. A major target of CLKs, SRSF4 (SRp75) is known to undergo dramatic changes in phosphorylation upon CLK inhibition and treatment with the CLK inhibitor T025 reduces pSRp75 levels in MDA-MB-468 xenograft mice models [27,28]. To validate CLK inhibition in cellulo, western analysis was performed, and the results indicate a loss in p-SRp75 levels upon **HSDs1400/1791** treatment in Caki-1 cells (Figure 7C). Moreover, the positive control, cirtuvivint also led to p-SRp75 reduction (SI, Figure S4) [28]. After confirming CLK inhibition in cellulo, we were motivated to assess the antiproliferative activity of the lead compounds against other cancer cell lines. Leukemia cell lines were selected as the NCI database indicated they have relatively abundant CLK1 mRNA expression; moreover, ongoing studies have revealed that CLK inhibition can have promising benefits against acute myeloid leukemia (AML) [29]. These preliminary findings motivated us to test the lead compounds against AML and we were excited to observe that the compounds were active against the Molm-14 cell line along with the quizartinib-resistant clones, Molm-14 D835Y (Figure 7A) [30].

Previous studies have shown that the inhibition of SR protein phosphorylation by CLK inhibitors mediates antiproliferative effects in cancer cells including human colorectal and melanoma cell lines [15,26]. Loss in CLK activity modulates mainly skipped exon type of AS leading to inhibition of cancer cell growth [15]. TG003, an inhibitor of CLK 1 and 4, is known to suppress cell cycle progression and induces G2 arrest in MCF7 cells [28]. Moreover, the CLK inhibitor T3 is known to promote G2/M arrest in HCT116 [31]. Additionally, a preliminary study has suggested that inhibition of ROCK signaling promotes cell cycle arrest in hepatocellular carcinoma [32]. Given the lead compounds are potent CLK/ROCKi, we investigated whether they could promote cell cycle arrest. Flow cytometric analysis using propidium iodide (PI) staining was used to obtain the proportion of cells at the various stages of cell cycle. Preliminary study on **HSD1400** treated Caki-1 revealed **HSD1400** induces G2/M arrest in Caki-1. Subsequently, **HSD1791** was observed to induce Caki-1 cell cycle arrest (Figure 7B). Next, we delved into the underlying mechanism of cell cycle arrest induction by the selective CLK inhibitor, **HSD1791**. CLK inhibition alters gene expression of key proteins involved in cell growth [33]. Since the compounds induce a reduction in the G1 population, we assessed the expression of the classical cell cycle regulator of the G1 phase, *cyclin d1* [20]. qPCR analysis of **HSD1791** treated Caki-1 indicates that *cyclin d1* mRNA levels are downregulated upon following 24 h compound treatment (SI, Figure S5). G1 progression is dependent on Rb, the downstream target of cyclin D1/CDK4/6; hence, phosphorylation of Rb protein (a crucial indicator of its activity) was analyzed upon **HSD1791** treatment (Figure 7D). Western analysis indicates pRB levels are lowered upon 24 h compound treatment, indicating increased Rb activity that leads to induction of cell cycle arrest.

## 3. Discussion

Compounds with polypharmacology have the potential to display enhanced efficacy and resolve resistance issues as it is more difficult for cancer cells to simultaneously develop resistance at multiple targets than at single targets. This study provides an example of simultaneous targeting of two kinase classes, CLK and ROCK, which have been shown to play key roles in cancer, and hence, dual targeting could enhance cancer cell killing. Several independent studies have reported that CLKs (that regulate cellular events such as cell growth and alternative splicing) and ROCK1/2 (that play key roles in cell migration) are emerging as potential anticancer targets [12,20]. For instance, dysregulated CLK expression has been observed across several solid tumor types as well as hematological malignancies [20]. Although increased CLK1 expression corresponds to poor disease prognosis in RCC (with advanced stage of the disease considered “lethal” due to very poor five-year survival rates) and upregulation of SR proteins (major targets of CLKs) is associated with renal carcinogenesis, unfortunately, antitumor effects of CLKi have not yet been characterized against RCC [19,20]. Regarding the current KIs against CLK/ROCK, very few potent ROCKi have been described in the literature; also, none of the CLKis have yet received FDA approval [15]. To address these multifold challenges, this study has identified novel, potent CLK/ROCKi synthesized via Povarov/Doebner-type MCR as promising anticancer therapeutics. Additionally, this study demonstrated that the “magic methylation” principle can be applied to achieve CLK selectivity. The lead compounds potently inhibit the RCC cell line, Caki-1 proliferation (IC_50_ 204–309 nM) compared to previously reported Caki cell line IC_50_ for the current standard of care against renal cancer, for instance, 5 µM and 8 µM for sunitinib and sorafenib, respectively [34]. The compounds also exhibited higher potency in Caki-1 compared to the CLKi, TG003 (Caki-1 IC_50_ > 30 µM) [35]. Also, there is a growing interest in the benefits of targeting CLK against AML, where disease relapse is frequent with poor survival rates in relapsed patients (<10% survival at 3 years) [36]. Around one-third of AML patients carry FLT3 duplication mutations; therefore, the results of this study are significant as the lead compounds are active against Molm-14 cell lines that carry FLT3 internal tandem duplication mutations [37]. Moreover, we have gained mechanistic insight into the suppression of CLK activity via docking analyses and in vitro target engagement. Aligning with preliminary studies that indicate CLK inhibition induces DNA damage, this study demonstrated that the top compounds promote DNA damage via p-H2AX upregulation [20,24]. Also, flow cytometry results indicated the lead candidate’s induced cell cycle arrest. Additionally, qPCR and Western analysis provided the mechanistic rationale behind the antiproliferative effects of the compounds, i.e., via downregulation of the cylinD/Rb pathway.

Delving deeper into the mechanistic significance of this study, there is a clear correlation of dysregulated activity of SR proteins (major downstream targets of CLKs) contributing to increased oncogenic splice variants such as *cyclin d1* and *vegf* transcripts across several cancers [38]. Given that loss of VHL activity occurs in >90% of sporadic clear cell RCC development due to dysregulated VEGFR signaling, and the current limitations of VEGFRi (including drug-induced chemoresistance and toxicity issues), we provide a novel approach to potentially target VEGR signaling by downregulating SR activity via CLK inhibition [18,39]. To gain further mechanistic insight, future studies will therefore focus on transcriptomic and proteomic analysis of the lead compounds to identify differential expression of key oncogenic targets at various stages of tumor development including angiogenesis, cell migration and metastasis.

## 4. Materials and Methods

### 4.1. General Synthetic Information

Unless noted otherwise, all reagents and solvents were purchased from commercial suppliers and used as received. All reactions were performed in a screw-cap 20 mL glass vial. The ^1^H and ^13^C NMR spectra were obtained in Methanol-*d*_4_ or DMSO-*d*_6_ as solvent using a 500 MHz spectrometer. Tetramethylsilane was used as an internal standard. Flash or column chromatography using silica gel (230−400 mesh) methods were utilized for purification. Coupling constants (*J* values) reported in Hz. All compounds were characterized by ^1^H and ^13^C NMR, and HRMS data. High-resolution mass spectra (HRMS) were recorded using electron spray ionization (ESI) and TOF mass analyzer techniques.

#### General Procedure for the Synthesis of Analogs

To a 20 mL reaction vial, charged with the corresponding amine (0.5 equivalent) and aldehyde (0.5 equivalent), we added 5 mL ethanol and refluxed it for an hour. After that, the reaction was cooled down to room temperature and the corresponding cyclic ketone (3 equivalent) was added, which was followed by the addition of a catalytic amount of concentrated hydrochloric acid; the reaction was allowed to reflux for another 8 to 12 h after the completion reaction mixture was concentrated and purified over silica gel chromatography using ethyl acetate/hexanes (80:20) or methanol/dichloromethane (10:90) as solvent system to obtain the desired product.

7-Phenyl-8,9,10,11-tetrahydro-3*H*-pyrazolo[4,3-*a*]phenanthridine (1); see reference for the experimental details [11].

7-(2-Methoxyphenyl)-8,9,10,11-tetrahydro-3*H*-pyrazolo[4,3-*a*]phenanthridine (2); see reference for the experimental details [11].

(3-(8,9,10,11-Tetrahydro-3*H*-pyrazolo[4,3-*a*]phenanthridin-7-yl)phenyl)boronic acid (3); see reference for the experimental details [11].

(4-(8,9,10,11-Tetrahydro-3*H*-pyrazolo[4,3-*a*]phenanthridin-7-yl)phenyl)boronic acid (4); see reference for the experimental details [11].

8,9,10,11-Tetrahydro-3*H*-pyrazolo[4,3-*a*]phenanthridine(5); see reference for the experimental details [11].

(2-Methoxy-3-(8,9,10,11-tetrahydro-3*H*-pyrazolo[4,3-*a*]phenanthridin-7-yl)phenyl)boronic acid (6)

Off-white solid (41 mg, 11%).^1^H NMR (500 MHz, Methanol-*d*_4_) δ 8.58 (s, 1H), 7.89 (d, *J* = 9.2 Hz, 1H), 7.83 (d, *J* = 9.1 Hz, 1H), 7.42 (d, *J* = 7.3 Hz, 1H), 7.32 (d, *J* = 7.4 Hz, 1H), 7.24 (t, *J* = 7.4 Hz, 1H), 3.48 (s, 3H), 3.44–3.35 (m, 2H), 2.82 (dd, *J* = 15.9, 7.2 Hz, 1H), 2.51 (dt, *J* = 17.3, 5.9 Hz, 1H), 2.09–2.00 (m, 2H), 1.89–1.82 (m, 1H), 1.81–1.76 (m, 1H); ^13^C NMR (126 MHz, Methanol-*d*_4_) δ 159.79, 155.68, 142.96, 133.21, 132.37, 131.13, 130.91, 128.28, 124.23, 123.00, 122.24, 29.48, 27.03, 22.16, 21.79. HRMS (ESI) m/z calculated for C_21_H_21_ BN_3_O_3_ [M + H]^+^ 374.1676, found 374.1670.

(2-Methoxy-3-((8*S*,11*R*)-8,9,10,11-tetrahydro-3*H*-8,11-methanopyrazolo[4,3-*a*]phenanthridin-7-yl)phenyl)boronic acid (7)

Off-white solid (46 mg, 24%). ^1^H NMR (500 MHz, Methanol-*d*_4_) δ 8.92 (s, 1H), 8.21 (d, *J* = 8.9 Hz, 1H), 8.05 (d, *J* = 9.0 Hz, 1H), 7.61 (d, *J* = 7.3 Hz, 2H), 7.35 (t, *J* = 7.3 Hz, 1H), 4.56 (d, *J* = 4.0 Hz, 1H), 3.64 (s, 3H), 3.57 (s, 1H), 2.41 (d, *J* = 11.8 Hz, 1H), 2.17 (t, *J* = 6.5 Hz, 1H), 2.11 (d, *J* = 9.2 Hz, 1H), 1.91 (d, *J* = 9.2 Hz, 1H), 1.40 (d, *J* = 8.5 Hz, 2H); ^13^C NMR (126 MHz, Methanol-*d*_4_) δ 160.89, 159.99, 143.34, 143.10, 138.29, 136.20, 132.55, 131.84, 131.22, 123.83, 123.27, 122.90, 121.02, 120.52, 117.93, 59.64, 49.62, 44.99, 42.24, 25.46, 24.28; HRMS (ESI) m/z calculated for C_22_H_21_BN_3_O_3_ [M + H] ^+^: 386.1676, found: 386.1681.

(2-Methoxy-5-(8,9,10,11-tetrahydro-3*H*-pyrazolo[4,3-*a*]phenanthridin-7-yl)phenyl)boronic acid (8); see reference for the experimental details [11].

(2-Methoxy-3-(9-methyl-3*H*-pyrazolo[4,3-*f*]quinolin-7-yl)phenyl)boronic acid (9); see reference for the experimental details [11].

(2-Methoxy-3-(9-(pyridin-4-yl)-3*H*-pyrazolo[4,3-*f*]quinolin-7-yl)phenyl)boronic acid (10); see reference for the experimental details [11].

(3-(5-Fluoro-8,9,10,11-tetrahydro-3*H*-pyrazolo[4,3-*a*]phenanthridin-7-yl)-2-methoxyphenyl)boronic acid (11)

Off-white solid (51 mg, 26%). ^1^H NMR (500 MHz, Methanol-*d*_4_) δ 8.86 (s, 1H), 8.05 (d, *J* = 10.1 Hz, 1H), 7.62 (d, *J* = 7.4 Hz, 1H), 7.57–7.50 (m, 1H), 7.35 (t, *J* = 7.4 Hz, 1H), 3.70 (s, 3H), 3.59 (t, *J* = 6.4 Hz, 2H), 2.77 (t, *J* = 6.0 Hz, 2H), 2.22–2.11 (m, 2H), 1.98–1.88 (m, 2H); ^13^C NMR (126 MHz, Methanol-*d*_4_) δ 159.70, 153.63, 153.08 (d, *J* = 253.2 Hz), 151.28, 137.61, 136.20, 134.65, 131.71, 131.06, 126.84, 126.70, 124.26, 123.85, 122.99, 122.62, 103.46, 59.30, 30.79, 27.06, 21.24, 20.88; HRMS (ESI) m/z calculated for C21H20BFN3O3 [M + H] ^+^: 392.1582, found: 392.1598.

(3-(5-Fluoro-1-methyl-8,9,10,11-tetrahydro-3*H*-pyrazolo[4,3-*a*]phenanthridin-7-yl)-2-methoxyphenyl)boronic acid (12, HSD1791)

Off-white solid (39 mg, 19%). ^1^H NMR (500 MHz, Methanol-*d*_4_) δ 7.47–7.38 (m, 2H), 7.32 (d, *J* = 7.4 Hz, 1H), 7.24 (t, *J* = 7.3 Hz, 1H), 3.53–3.43 (m, 5H), 2.91 (s, 3H), 2.87–2.79 (m, 1H), 2.58–2.46 (m, 1H), 1.95–1.73 (m, 4H); ^13^C NMR (126 MHz, Methanol-*d*_4_) δ 159.72, 158.15 (d, *J* = 253.2 Hz), 155.86, 142.91, 135.37, 134.59, 133.21, 132.69, 132.48, 132.23, 131.47, 131.18, 125.08, 123.39, 122.99, 120.47, 60.13, 60.10, 31.88, 26.75, 22.08, 21.66; HRMS (ESI) m/z calculated for C_22_H_22_BFN_3_O_3_ [M + H] ^+^: 406.1738, found: 406.1752.

(2-Methoxy-3-(1-methyl-8,9,10,11-tetrahydro-3*H*-pyrazolo[4,3-*a*]phenanthridin-7-yl)phenyl)boronic acid (13)

Off-white solid (76 mg, 39%). ^1^H NMR (500 MHz, Methanol-*d*_4_) δ 8.16 (d, *J* = 9.2 Hz, 1H), 7.89 (d, *J* = 9.2 Hz, 1H), 7.64 (dd, *J* = 7.4, 1.8 Hz, 1H), 7.53 (dd, *J* = 7.5, 1.7 Hz, 1H), 7.36 (t, *J* = 7.5 Hz, 1H), 3.80–3.75 (m, 2H), 3.69 (s, 3H), 3.05 (s, 3H), 2.83–2.78 (m, 2H), 2.02–1.93 (m, 4H); ^13^C NMR (126 MHz, Methanol-*d*_4_) δ 159.58, 154.27, 148.73, 137.76, 136.35, 135.67, 132.68, 130.83, 126.09, 124.38, 123.13, 122.68, 119.12, 112.78, 59.25, 33.00, 26.44, 21.26, 20.70; HRMS (ESI) m/z calculated for C_22_H_23_BN_3_O_3_ [M + H] ^+^: 388.1832, found: 388.1840.

2-Methoxy-3-(8,9,10,11-tetrahydro-3*H*-pyrazolo[4,3-*a*]phenanthridin-7-yl)benzoic acid (14)

Off-white solid (571 mg, 65%). Its triflate salt as trifluoroacetic acid was used to isolate from column chromatography. ^1^H NMR (500 MHz, DMSO-*d*_6_) δ 8.79 (s, 1H), 8.17 (d, *J* = 9.1 Hz, 1H), 8.03 (d, *J* = 9.2 Hz, 1H), 7.92 (dd, *J* = 7.8, 1.7 Hz, 1H), 7.67 (d, *J* = 7.5 Hz, 1H), 7.41 (t, *J* = 7.7 Hz, 1H), 3.54–3.40 (m, 5H), 2.72–2.53 (m, 2H), 2.07–1.95 (m, 2H), 1.88–1.73 (m, 2H); ^13^C NMR (126 MHz, DMSO-*d*_6_) δ 167.41, 158.73 (q, J = 35.2 Hz), 157.15, 134.60, 133.15, 131.88, 126.69, 124.39, 123.26, 115.51 (q, *J* = 294.8 Hz), 115.40, 62.73, 30.38, 27.30, 22.03, 21.57. HRMS (ESI) m/z calculated for C_22_H_20_BN_3_O_3_ [M + H] ^+^: 374.1505, found: 374.1507.

7-(2,3-Dimethoxyphenyl)-8,9,10,11-tetrahydro-3*H*-pyrazolo[4,3-*a*]phenanthridine (15)

Off-white solid (101 mg, 56%). ^1^H NMR (500 MHz, DMSO-*d*_6_) δ 7.80 (s, 1H), 7.14–7.00 (m, 2H), 6.41 (t, *J* = 7.9 Hz, 1H), 6.35 (d, *J* = 8.2 Hz, 1H), 6.06 (d, *J* = 7.5 Hz, 1H), 3.12 (s, 3H), 2.76 (s, 3H), 2.69–2.57 (m, 2H), 1.95 (dd, *J* = 16.2, 8.0 Hz, 1H), 1.73 (dd, *J* = 14.8, 8.4 Hz, 1H), 1.30–1.22 (m, 2H), 1.11–0.93 (m, 2H); ^13^C NMR (126 MHz, DMSO-*d*_6_) δ 154.49, 152.12, 145.72, 142.09, 141.90, 137.88, 135.03, 134.07, 130.08, 127.50, 123.39, 121.44, 120.58, 115.42, 112.75, 111.91, 59.11, 54.20, 28.70, 26.27, 21.36, 20.99; HRMS (ESI) m/z calculated for C_22_H_22_BN_3_O_2_ [M + H] ^+^: 360.1712, found: 360.1715.

7-(2-Methoxypyridin-3-yl)-8,9,10,11-tetrahydro-3*H*-pyrazolo[4,3-*a*]phenanthridine (16); see reference for the experimental details [11].

(5-Fluoro-2-methoxy-3-(8,9,10,11-tetrahydro-3*H*-pyrazolo[4,3-*a*]phenanthridin-7-yl)phenyl)boronic acid (17)

Off-white solid (83 mg, 29%). ^1^H NMR (500 MHz, DMSO-*d*_6_) δ 8.56 (s, 1H), 8.20 (s, 2H), 7.83 (q, *J* = 9.1 Hz, 2H), 7.26 (dd, *J* = 8.6, 3.3 Hz, 1H), 7.11 (dd, *J* = 8.5, 3.3 Hz, 1H), 3.39–3.33 (m, 5H), 2.74–2.63 (m, 1H), 2.55–2.50 (m, 1H), 2.01–1.91 (m, 2H), 1.80–1.71 (m, 2H); ^13^C NMR (126 MHz, DMSO-*d*_6_) δ 158.95 (d, *J* = 241.9 Hz), 157.20, 154.54, 143.38, 141.90, 138.73, 136.33, 135.74, 131.00, 130.20, 129.59, 122.08, 120.15 (d, *J* = 20.9 Hz), 118.29 (d, *J* = 23.9 Hz), 116.52, 114.29, 61.99, 29.51, 27.46, 22.62, 22.18; HRMS (ESI) m/z calculated for C_21_H_20_BFN_3_O_3_ [M + H] ^+^: 392.1582, found: 392.1593.

(2-Methoxy-5-methyl-3-(8,9,10,11-tetrahydro-3*H*-pyrazolo[4,3-*a*]phenanthridin-7-yl)phenyl)boronic acid (18)

Off-white solid (108 mg, 38%). ^1^H NMR (500 MHz, Methanol-*d*_4_) δ 8.87 (s, 1H), 8.29 (d, *J* = 9.2 Hz, 1H), 8.04 (d, *J* = 9.2 Hz, 1H), 7.45 (s, 1H), 7.37 (s, 1H), 3.68–3.57 (m, 5H), 2.90–2.73 (m, 2H), 2.43 (s, 3H), 2.18 (p, *J* = 6.0 Hz, 2H), 1.94 (p, *J* = 6.0 Hz, 2H); ^13^C NMR (126 MHz, Methanol-*d*_4_) δ 157.58, 154.14, 149.51, 138.42, 136.76, 134.68, 133.30, 133.01, 132.66, 131.87, 131.17, 124.81, 123.77, 123.12, 119.10, 114.91, 59.54, 30.77, 26.81, 21.34, 20.93, 19.15; HRMS (ESI) m/z calculated for C_22_H_23_BN_3_O_3_ [M + H] ^+^: 388.1832, found: 388.1844.

(3-(9,9-Dioxido-3,8,10,11-tetrahydropyrazolo[4,3-*f*]thiopyrano[3,4-*c*]quinolin-7-yl)-5-fluoro-2-methoxyphenyl)boronic acid (19)

Off-white solid (10 mg, 11%). ^1^H NMR (500 MHz, DMSO-*d*_6_) δ 8.66 (s, 1H), 8.34 (s, 2H), 7.99 (d, *J* = 9.1 Hz, 1H), 7.90 (d, *J* = 9.0 Hz, 1H), 7.30 (dd, *J* = 8.5, 3.2 Hz, 1H), 7.11 (dd, *J* = 8.4, 3.2 Hz, 1H), 4.40–4.35 (m, 1H), 4.22 (d, *J* = 15.7 Hz, 1H), 4.01 (q, *J* = 6.7 Hz, 2H), 3.80–3.71 (m, 2H), 3.68–3.60 (m, 1H), 3.38 (s, 3H), 2.07 (s, 1H). ^13^C NMR (126 MHz, DMSO-*d*_6_) δ 158.96, 157.04, 156.51, 153.81, 144.49, 139.02, 138.22, 136.65, 133.70, 129.47, 123.52, 121.24, 118.31, 118.12, 116.04, 61.93, 50.92, 45.96, 40.49, 40.41, 40.32, 40.15, 39.99, 39.82, 39.65, 39.49, 31.17, 30.60. HRMS (ESI) m/z calculated for C_20_H_17_BFN_3_O_5_S [M + H] ^+^: 442.10442, found: 442.10411.

(5-Fluoro-2-methoxy-3-(1-methyl-9,9-dioxido-3,8,10,11-tetrahydropyrazolo[4,3-*f*]thiopyrano[3,4-*c*]quinolin-7-yl)phenyl)boronic acid (20)

Off-white solid (15 mg, 13%). ^1^H NMR (500 MHz, DMSO-*d*_6_) δ 8.35 (s, 2H), 7.87 (d, *J* = 9.0 Hz, 1H), 7.80 (d, *J* = 9.2 Hz, 1H), 7.29 (dd, *J* = 8.4, 3.3 Hz, 1H), 7.23–7.16 (m, 1H), 4.52 (d, *J* = 15.6 Hz, 1H), 4.21 (d, *J* = 15.5 Hz, 1H), 4.05 (d, *J* = 28.3 Hz, 2H), 3.54 (d, *J* = 13.5 Hz, 1H), 3.34 (s, 3H), 3.17 (d, *J* = 27.9 Hz, 1H), 2.81 (s, 3H), 2.07 (s, 1H). ^13^C NMR (126 MHz, DMSO-*d*_6_) δ 159.00, 157.07, 156.62, 152.73, 145.35, 143.19, 141.30, 140.30, 133.21, 131.85, 128.88, 123.68, 122.44, 121.43, 117.60, 117.22, 115.88, 114.36, 62.48, 61.34, 48.73, 40.50, 40.33, 40.17, 40.00, 39.83, 39.67, 39.50, 39.38, 30.67, 28.97. HRMS (ESI) m/z calculated for C_21_H_19_BFN_3_O_5_S [M + H] ^+^: 456.12007, found: 456.11958.

### 4.2. Cell Culture and Viability Assay

Molm 14 and Molm 14 D835Y were a generous gift from the laboratory of Dr Neil Shah, Department of Medicine, University of California, San Fransisco, CA, USA). Caki-1, ACHN and 786-O (purchased from ATCC, Manassas, VA, USA) were maintained as per the manufacturer’s protocol in a 5% CO2 incubator at 37 °C. Caki-1 cells were cultured in DMEM (10% FBS and penicillin-streptomycin), stock compounds were prepared in DMSO, and working concentrations of the compounds were obtained upon diluting the stock compounds in media. Cells were seeded in 96-well plates (2000 cells/well), after overnight incubation cells were treated with the compounds for 72 h. Post-treatment, cells were incubated with CellTiter-Blue Cell Viability Assay dye (Promega, Madison, WI, USA) for 3 h and the viability reading was taken in BioTek Cytation5 plate reader (Agilent, Santa Clara, CA, USA), according to the manufacturer’s protocol.

### 4.3. Kinase Assay

ADP-Glo assay was performed as previously described [40]. Original stock compound concentrations were prepared by dissolving the compounds in DMSO. On the day of the experiment, 5X compound stocks were prepared in kinase buffer (25 mM HEPES, 10 mM magnesium acetate, 1 mM DTT, 0.01% Bovine Serum Albumin and 0.01% Tween-20), from which 1X final working concentration was used in the reaction. The kinase reaction included 40 μM ATP, 0.16 mg/mL MBP and 3.2 ng/μL CLK1 enzyme and the mixture was incubated for 3 h at room temperature. Luminescence was recorded using a Biotek Cytation5 plate reader.

### 4.4. Docking Analysis

Protein kinases CLK1, PDB: 6QTY, and CLK4, PDB: 6FYV crystal structures were obtained from the RCSB Protein Data Bank, and were prepared using the Protein Preparation Wizard; Epik, Schrödinger, LLC, New York, NY, USA, 2023; Impact, Schrödinger, LLC, New York, NY, USA, 2023; Prime, Schrödinger, LLC, New York, NY, USA, 2023. Ligands HSD1400 and HSD1791 were written as mol2 files, transferred to Maestro, and prepared for docking using the LigPrep protocol, LigPrep, Schrödinger, LLC, New York, NY, USA, 2023. Maestro, Schrödinger, LLC, New York, NY, USA, 2023, was used to evaluate generated docking poses. The PyMol Molecular Graphics System, Version 2.0 Schrödinger, LLC, was used to produce visual representations.

### 4.5. Western Analysis

Caki-1 was seeded in 6-well plates (around 1 × 10^6^/well). After overnight incubation, cells were treated with the compounds. Post-treatment, cells were harvested in RIPA buffer. Post gel electrophoresis, transfer was performed at 80 V for 155 min. The nitrocellulose membrane was probed with pRb, Rb, actin and p-H2AX (Cell Signaling Technology, Danvers, MA, USA) and p-SR (MilliporeSigma, Burlington, MA, USA) antibodies

### 4.6. qPCR Assay

Around 1 × 10^6^ Caki-1 were seeded in 6-well plates and the assay was performed as previously described [41]. 1000 ng RNA was used for cDNA synthesis and the primers were used as previously reported [42,43]. qPCR cycle was performed in the CFX96 Real-Time System (Bio-Rd, Hercules, CA, USA). An unpaired *t*-test was used to assess statistical significance.

### 4.7. Flow Cytometry Assay

The assay was performed as previously described [43]. Briefly, around 1 × 10^6^ cells were seeded, and following overnight incubation, cells were treated with the respective compounds. Post-fixation with 70% ethanol, staining was performed at room temperature using FxCycle dye (ThermoFisher Scientific, Waltham, MA, USA) for 15 min after which analysis was performed in BD Accuri C6 (BD Biosciences, San Jose, CA, USA). Cell cycle analysis was carried out using FlowJo software. Statistical significance was determined using an unpaired *t*-test.

## 5. Conclusions

This study has identified novel pyrazolo quinoline-based compounds **HSD1400** and **HSD1791** as potent CLK/ROCKi via a simple one-pot Povarov/Doebner approach. The popular medicinal chemistry principle of “magic methylation” was used to derive **HSD1791** from **HSD1400**; therefore, a novel approach to tune CLK selectivity has been demonstrated that can be utilized to develop more potent, drug-like analogs. The lead compounds suppress CLK1 activity in vitro and promote DNA damage via p-H2AX upregulation. Also, the compounds inhibit Caki-1 cell proliferation and are also active against leukemia cell lines. Furthermore, the lead compounds induce cell cycle arrest in Caki-1. Mechanistically, qPCR analysis revealed **HSD1791** lowered *cyclin D1* expression in Caki-1 cells. Furthermore, phosphorylation levels of the cyclin D1 target, Rb, were reduced upon **HSD1791** treatment, indicating induction of cell cycle arrest via modulation of cyclin D/Rb pathway.

## Data Availability

Data is contained within the article and Supplementary Materials.

## Supplementary Materials

The following supporting information can be downloaded at: https://www.mdpi.com/article/10.3390/ph17121660/s1, Figure S1: A-C ADP-Glo assay, CTX-712 kept as positive control (90.2% inhibition at 50 nM) D. Caki-1 viability at 72h drug treatment; Figure S2: A. HSD1400 docked to CLK4. B. HSD1791 docked to CLK4; Figure S3: Compounds docked to CLK1 (PDB: 6QTY); Table S1: Viability of renal cancer cell lines at 72 h drug treatment; Figure S4: Western blotting analysis, Caki-1 treated with 500 nM cirtuvivint for 24 h; Figure S5: Relative level of *cyclin d1* mRNA upon HSD1791 treatment; Figure S6: Full blots of western analysis; Table S2: Kinase profiling of lead candidate compounds tested at 25 nM, results provided by Reaction Biology; Table S3: Kinome profiling of HSD1995 (tested at 100 nM) against 371 kinases, results provided by Reaction Biology; Table S4: Kinome profiling of HSD1400 (tested at 500 nM) against 125 kinases, results provided by Reaction Biology; Table S5: Docking scores of potent and non-potent CLK1 inhibitors.
