# Supplementary material for: Boronic Acid-Containing 3H- pyrazolo[4,3-f]quinoline Compounds as Dual CLK/ROCK Inhibitors with Anticancer Properties
Source: Pharmaceuticals (Basel). 2024 Dec 10;17(12):1660. doi: 10.3390/ph17121660 (PMC11677846; doi:10.3390/ph17121660)

SUPPLEMENTARY INFORMATION

Boronic acid-containing 3*H*- pyrazolo[4,3-*f*]quinoline compounds as dual CLK/ROCK inhibitors with anticancer properties

Neetu Dayal[1,2,†], Riddhi Chaudhuri[1,2,†], Kofi Simpa Yeboah[1,2], Nickolas R. Brauer[1,2] and Herman O. Sintim*[1,2,3,4]

[1]*Department of Chemistry, Purdue University, 560 Oval Drive, West Lafayette, IN 47907, USA*
[2]*Purdue Institute for Drug Discovery, Purdue University, 720 Clinic Drive, West Lafayette, IN 47907, USA*
[3]*Purdue Institute for Cancer Research, Purdue University, 201 S. University Street, West Lafayette, IN 47907, USA*
[4]*Current: Department of Chemistry and Biochemistry, University of Notre Dame, 251 Nieuwland Science Hall Notre Dame, IN 46556, USA*

†*These authors contributed equally to this work; *Corresponding author, e-mail: hsintim@nd.edu*

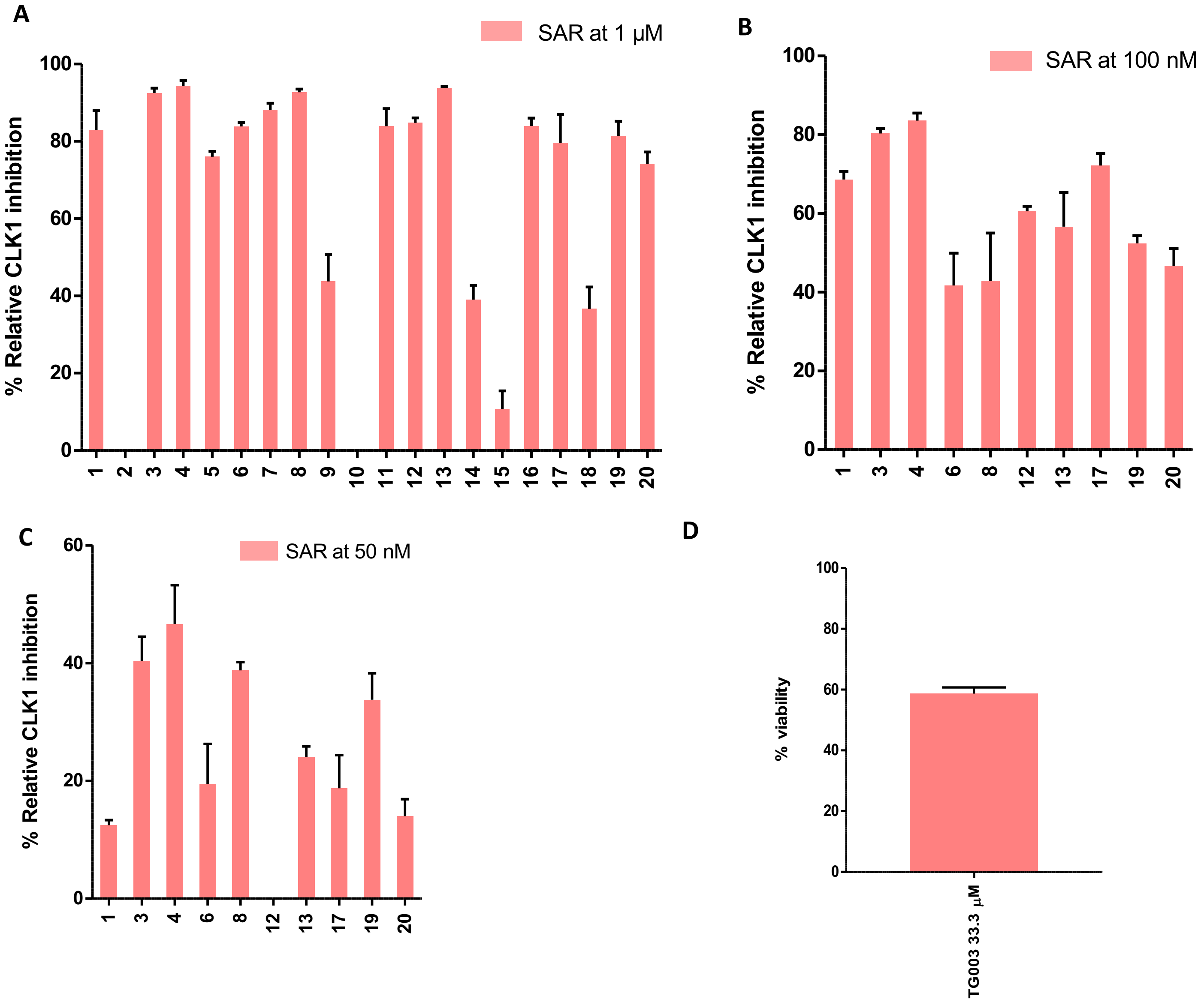

Figure S1: A-C ADP-Glo assay, CTX-712 kept as positive control (90.2% inhibition at 50 nM) D. Caki-1 viability at 72h drug treatment.

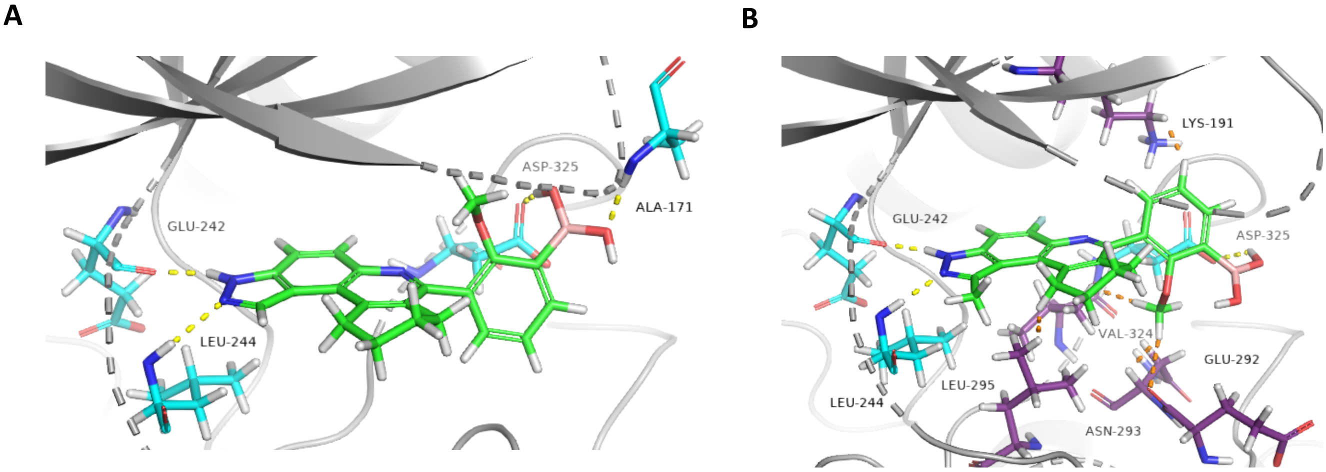

Figure S2: A. **HSD1400** docked to CLK4. B. **HSD1791** docked to CLK4. Yellow dashes represent hydrogen bonding interactions, orange represent clashes.

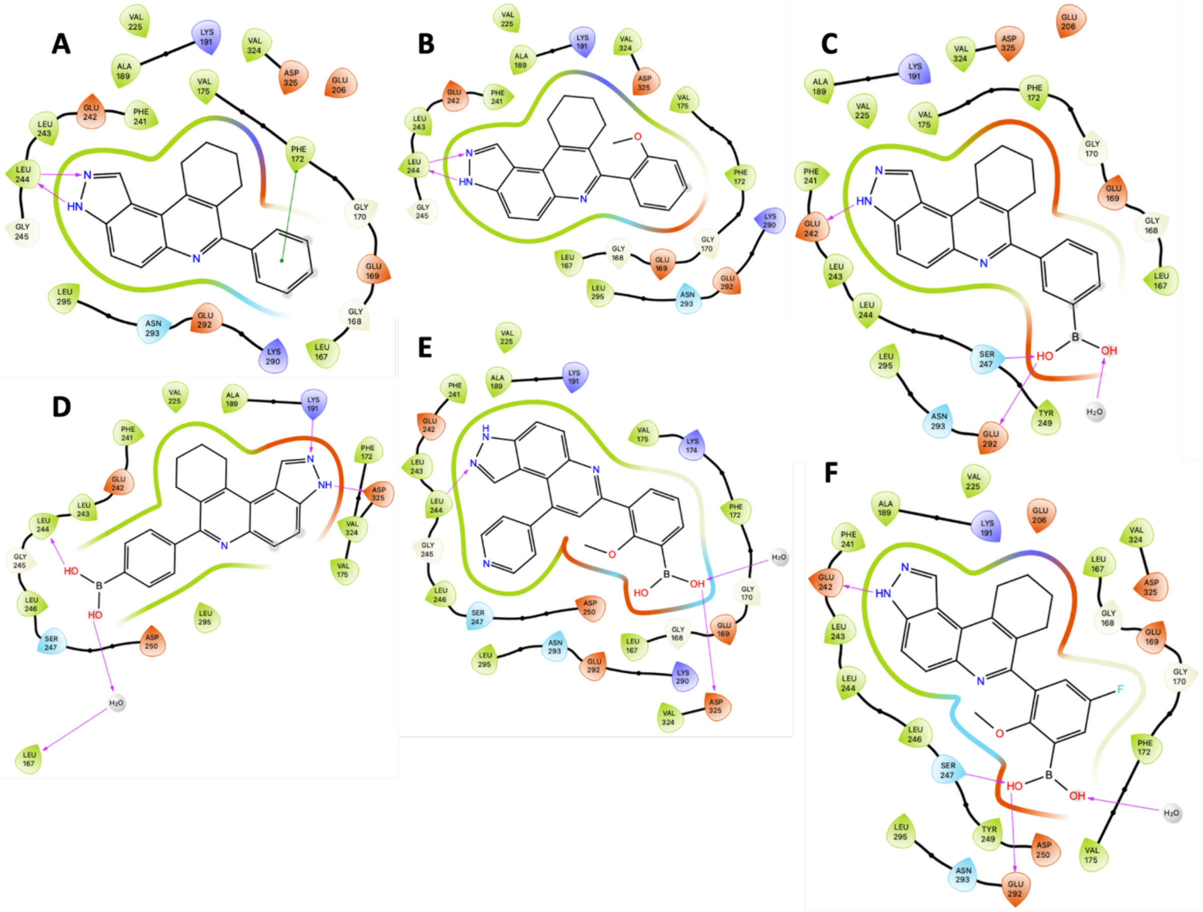

Figure S3: Compounds docked to CLK1 (PDB: 6QTY). A. Compound **1**. B. Compound **2**. C. Compound **3**. D. Compound **4**. E. Compound **10**. F. **HSD1995**.

Table S1: Viability of renal cancer cell lines at 72h drug treatment.

Compound	% ACHN inhibition		% 786-O inhibition	
	@ 1 µM	@ 10 µM	@ 1 µM	@ 10 µM
**3**	57	65	26	71
**4**	41	55	16	65
**6**	44	82	44	94
**8**	24	74	18	14
**12**	39	85	26	68
**13**	43	88	32	81
**17**	43	83	42	94
**19**	20	32	10	59

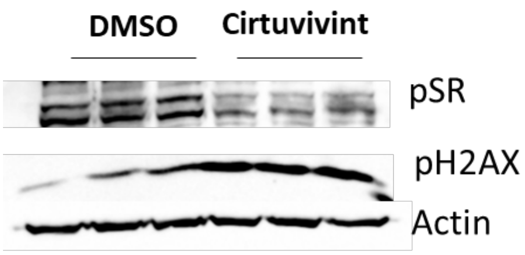

Figure S4: Western blotting analysis, Caki-1 treated with 500 nM cirtuvivint for 24 h.

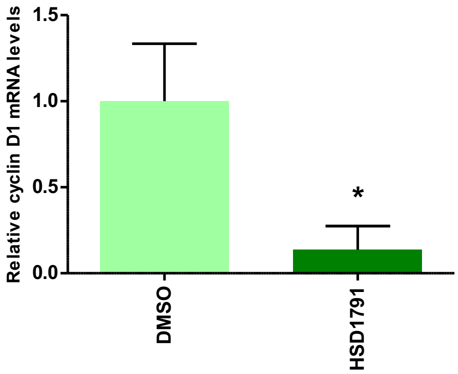

Figure S5: Relative level of *cyclin d1* mRNA (qPCR) upon HSD1791 treatment of Caki-1. Assay included 4 replicates per group. Error bar represents mean ± SD. * represents p<0.05.

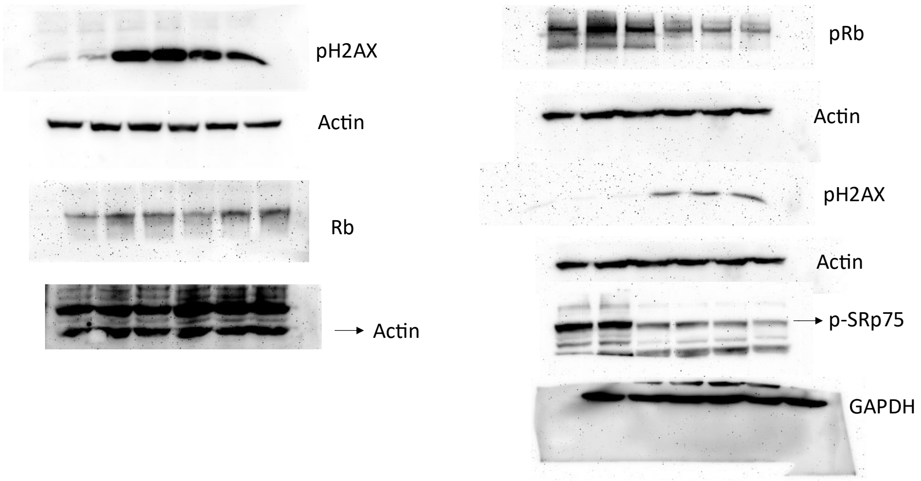

Figure S6: Full blots of western analysis.

Table S2: Kinase profiling of lead candidates, compounds tested at 25 nM, results provided by Reaction Biology.

	Percent enzyme inhibition of compounds		
**kinase**	**HSD1400**	**HSD1995**	**HSD1791**
CLK1	99	47	85
CLK2	96	52	78
CLK4	84	0	0
CDK12	39	22	0
CDK13	8	41	n.d
CDK14	91	98	9
CDK16	75	88	n.d
CDK17	79	93	n.d
CDK18	95	98	18
MSK2	98	59	45
*CK2a	n.d	59	9
*ROCK1	94	98	n.d
ROCK2	99	99	70

* indicates CK2a tested at 250 nM, while ROCK1 at 100 nM (**HSD1995**) and 500 nM (**HSD1400**), n.d is not determined

Table S3: Kinome profiling of **HSD1995** (tested at 100 nM) against 371 kinases, results provided by Reaction Biology.

Kinase	% Inhibition
ABL1	1
ABL2/ARG	3
ACK1	2
AKT1	19
AKT2	15
AKT3	19
ALK	0
ALK1/ACVRL1	15
ALK2/ACVR1	8
ALK3/BMPR1A	4
ALK4/ACVR1B	8
ALK5/TGFBR1	3
ALK6/BMPR1B	5
ARAF	0
ARK5/NUAK1	17
ASK1/MAP3K5	0
AURORA A	27
AURORA B	20
AURORA C	6
AXL	22

BLK	13
BMPR2	9
BMX/ETK	13
BRAF	8
BRK	0
BRSK1	21
BRSK2	31
BTK	9
C-KIT	22
C-MER	1
C-MET	9
C-SRC	6
CAMK1A	2
CAMK1B	4
CAMK1D	7
CAMK1G	3
CAMK2A	42
CAMK2B	27
CAMK2D	33
CAMK2G	5
CAMK4	0
CAMKK1	3
CAMKK2	18
CDC7/DBF4	0
CDK1/CYCLIN A	46
CDK1/CYCLIN B	51
CDK1/CYCLIN E	41
CDK14/CYCLIN Y (PFTK1)	97
CDK16/CYCLIN Y (PCTAIRE)	95
CDK17/CYCLIN Y (PCTK2)	91
CDK18/CYCLIN Y (PCTK3)	98
CDK19/CYCLIN C	13
CDK2/CYCLIN A	38
CDK2/CYCLIN A1	52
CDK2/CYCLIN E	50
CDK2/CYCLIN E2	58
CDK2/CYCLIN O	56
CDK3/CYCLIN E	75
CDK3/CYCLIN E2	79
CDK4/CYCLIN D1	1
CDK4/CYCLIN D3	7
CDK5/P25	69
CDK5/P35	82

CDK6/CYCLIN D1	8
CDK6/CYCLIN D3	64
CDK7/CYCLIN H	68
CDK8/CYCLIN C	11
CDK9/CYCLIN K	37
CDK9/CYCLIN T1	66
CDK9/CYCLIN T2	74
CHK1	7
CHK2	6
CK1A1	3
CK1A1L	0
CK1D	0
CK1EPSILON	7
CK1G1	4
CK1G2	11
CK1G3	7
CK2A	41
CK2A2	47
CLK1	95
CLK2	89
CLK3	27
CLK4	87
COT1/MAP3K8	6
CSK	0
CTK/MATK	3
DAPK1	1
DAPK2	2
DCAMKL1	18
DCAMKL2	20
DDR1	14
DDR2	18
DMPK	0
DMPK2	73
DRAK1/STK17A	5
DYRK1/DYRK1A	51
DYRK1B	54
DYRK2	22
DYRK3	25
DYRK4	0
EGFR	7
EPHA1	2
EPHA2	12
EPHA3	21
EPHA4	0
EPHA5	0
EPHA6	6

EPHA7	2
EPHA8	0
EPHB1	0
EPHB2	12
EPHB3	9
EPHB4	7
ERBB2/HER2	0
ERBB4/HER4	4
ERK1	0
ERK2/MAPK1	13
ERK5/MAPK7	7
ERK7/MAPK15	54
ERN1/IRE1	25
ERN2/IRE2	0
FAK/PTK2	14
FER	6
FES/FPS	0
FGFR1	7
FGFR2	0
FGFR3	0
FGFR4	14
FGR	6
FLT1/VEGFR1	6
FLT3	39
FLT4/VEGFR3	0
FMS	0
FRK/PTK5	4
FYN	11
GCK/MAP4K2	17
GLK/MAP4K3	7
GRK1	18
GRK2	5
GRK3	8
GRK4	12
GRK5	7
GRK6	32
GRK7	10
GSK3A	38
GSK3B	50
HASPIN	38
HCK	10
HGK/MAP4K4	7
HIPK1	7
HIPK2	0
HIPK3	5
HIPK4	11

HPK1/MAP4K1	14
IGF1R	0
IKKA/CHUK	0
IKKB/IKBKB	3
IKKE/IKBKE	10
IR	7
IRAK1	9
IRAK4	0
IRR/INSRR	22
ITK	2
JAK1	10
JAK2	19
JAK3	83
JNK1	11
JNK2	3
JNK3	16
KDR/VEGFR2	3
KHS/MAP4K5	0
KSR1	0
KSR2	0
LATS1	43
LATS2	63
LCK	0
LCK2/ICK	45
LIMK1	88
LIMK2	7
LKB1	27
LOK/STK10	0
LRRK2	71
LYN	5
LYN B	0
MAK	52
MAPKAPK2	8
MAPKAPK3	0
MAPKAPK5/PRAK	9
MARK1	10
MARK2/PAR-1BA	13
MARK3	12
MARK4	6
MAST3	11
MASTL	0
MEK1	0
MEK2	8
MEK3	0
MEK5	0
MEKK1	9

MEKK2	9
MEKK3	26
MEKK6	0
MELK	53
MINK/MINK1	5
MKK4	0
MKK6	5
MKK7	9
MLCK/MYLK	20
MLCK2/MYLK2	18
MLK1/MAP3K9	0
MLK2/MAP3K10	21
MLK3/MAP3K11	10
MLK4	0
MNK1	16
MNK2	24
MRCKA/CDC42BPA	18
MRCKB/CDC42BPB	22
MSK1/RPS6KA5	86
MSK2/RPS6KA4	95
MSSK1/STK23	31
MST1/STK4	3
MST2/STK3	20
MST3/STK24	18
MST4	19
MUSK	12
MYLK3	19
MYLK4	13
MYO3A	18
MYO3B	3
NEK1	0
NEK11	0
NEK2	3
NEK3	8
NEK4	0
NEK5	7
NEK6	6
NEK7	0
NEK9	7
NIM1	3
NLK	6
OSR1/OXSR1	4
P38A/MAPK14	8
P38B/MAPK11	9
P38D/MAPK13	1
P38G	0

P70S6K/RPS6KB1	88
P70S6KB/RPS6KB2	52
PAK1	0
PAK2	10
PAK3	14
PAK4	1
PAK5	11
PAK6	0
PASK	28
PBK/TOPK	9
PDGFRA	7
PDGFRB	16
PDK1/PDPK1	0
PHKG1	37
PHKG2	2
PIM1	54
PIM2	0
PIM3	3
PKA	16
PKACB	18
PKACG	3
PKCA	26
PKCB1	20
PKCB2	13
PKCD	70
PKCEPSILON	77
PKCETA	81
PKCG	2
PKCIOTA	17
PKCMU/PRKD1	0
PKCNU/PRKD3	16
PKCTHETA	38
PKCZETA	3
PKD2/PRKD2	0
PKG1A	78
PKG1B	75
PKG2/PRKG2	23
PKMYT1	34
PKN1/PRK1	76
PKN2/PRK2	16
PKN3/PRK3	45
PLK1	36
PLK2	0
PLK3	0
PLK4/SAK	23
PRKX	6

PYK2	11
RAF1	0
RET	17
RIPK2	30
RIPK4	0
RIPK5	0
ROCK1	98
ROCK2	100
RON/MST1R	0
ROS/ROS1	7
RSK1	81
RSK2	63
RSK3	63
RSK4	23
SBK1	0
SGK1	12
SGK2	8
SGK3/SGKL	4
SIK1	0
SIK2	0
SIK3	14
SLK/STK2	4
SNARK/NUAK2	15
SNRK	7
SRMS	0
SRPK1	28
SRPK2	18
SSTK/TSSK6	9
STK16	16
STK21/CIT	4
STK22D/TSSK1	38
STK25/YSK1	19
STK32B/YANK2	0
STK32C/YANK3	9
STK33	26
STK38/NDR1	30
STK38L/NDR2	28
STK39/STLK3	30
SYK	0
TAK1	0
TAOK1	74
TAOK2/TAO1	4
TAOK3/JIK	70
TBK1	10
TEC	0
TESK1	17

TESK2	0
TGFBR2	0
TIE2/TEK	1
TLK1	5
TLK2	9
TNIK	18
TNK1	0
TRKA	0
TRKB	14
TRKC	15
TSSK2	11
TSSK3/STK22C	13
TTBK1	6
TTBK2	0
TXK	2
TYK1/LTK	0
TYK2	8
TYRO3/SKY	0
ULK1	33
ULK2	11
ULK3	26
VRK1	0
VRK2	29
WEE1	11
WNK1	12
WNK2	9
WNK3	8
YES/YES1	8
YSK4/MAP3K19	33
ZAK/MLTK	5
ZAP70	3
ZIPK/DAPK3	25

Table S4: Kinome profiling of **HSD1400** (tested at 500 nM) against 125 kinases, results provided by Reaction Biology.

Kinase	% Inhibition
ABL1	0
ABL1 (T315I)	n.d
ABL2/ARG	6
AKT2	0
AKT3	2

ALK1/ACVRL1	0
ALK2/ACVR1	0
ALK3/BMPR1A	8
ALK4/ACVR1B	0
ALK5/TGFBR1	0
ALK6/BMPR1B	9
Aurora A	56
AURORA B	17
AURORA C	15
BMX/ETK	0
BRAF	0
BRK	2
c-Kit	0
c-MER	0
c-MET	0
c-Src	2
CAMKK2	23
CDK1/cyclin B	59
CDK14/cyclin Y (PFTK1)	87
CDK16/cyclin Y (PCTAIRE)	84
CDK17/cyclin Y (PCTK2)	79
CDK18/cyclin Y (PCTK3)	93
CDK19/cyclin C	37
CDK2/CYCLIN A	83
CDK2/cyclin E	57
CDK3/cyclin E	86
CDK4/cyclin D1	13
CDK5/P25	76
CDK6/cyclin D1	4
CDK7/cyclin H	66
CDK8/cyclin C	21
CDK9/cyclin T1	19
CHK1	0
CHK2	5
CK1a1	0
CLK1	91
CLK2	73
CLK3	41
CLK4	89
COT1/MAP3K8	0
DAPK1	2
DDR1	13
DDR2	0

EGFR	8
ERBB2/HER2	3
ERK5/MAPK7	3
ERK7/MAPK15	27
ERN1/IRE1	41
FAK/PTK2	0
FER	0
FGFR1	0
FGFR2	0
FGFR3	0
FGFR4	9
FGR	0
FLT3 (D835Y)	n.d
FLT3 (ITD)	n.d
FMS	0
FYN	0
GLK/MAP4K3	0
GSK3a	31
GSK3b	11
Haspin	52
HCK	0
HGK/MAP4K4	0
HPK1/MAP4K1	1
IGF1R	0
IRAK4	0
IRR/INSRR	0
ITK	11
JAK1	2
JAK2	3
JAK2 (V617F)	0
JAK3	25
KDR/VEGFR2	13
KSR1	4
KSR2	0
LCK	16
LIMK1	63
LIMK2	0
MNK1	12
MNK2	31
NEK2	0
p70S6K/RPS6KB1	85
PBK/TOPK	30
PDGFRa	1
PDGFRb	6
PDK1/PDHK1	0
PIM1	6

PIM2	0
PIM3	0
PLK1	35
PYK2	0
RET	11
RIPK3	32
RIPK4	20
ROCK1	94
RON/MST1R	3
RSK1	73
SGK1	13
SGK2	0
STK39/STLK3	43
SYK	15
TBK1	2
TRKA	6
TRKB	0
TRKC	31
TRPM7/CHAK1	6
TTK	7
TXK	0
TYK1/LTK	2
TYK2	3
TYRO3/SKY	0
ULK1	41
WEE1	5
WNK1	0
YES/YES1	0
ZAK/MLTK	5
ZAP70	8
ZIPK/DAPK3	0

Table S5: Docking scores of potent and non-potent CLK1 inhibitors. Docking scores for **HSD1400** and **HSD1791** correspond to Figure 5A. and 5B. Scores for compounds **1**, **2**, **3**, **4**, **10**, and **HSD1995** correspond to SI, Figure S6.

Compound	Docking Score
**HSD1400**	-11.69
**HSD1791**	-11.28
**1**	-11.41
**2**	-10.28
**3**	-10.59
**4**	-11.20
**10**	-10.06
**HSD1995**	-10.73

^{1}H and ^{13}C NMR Spectra

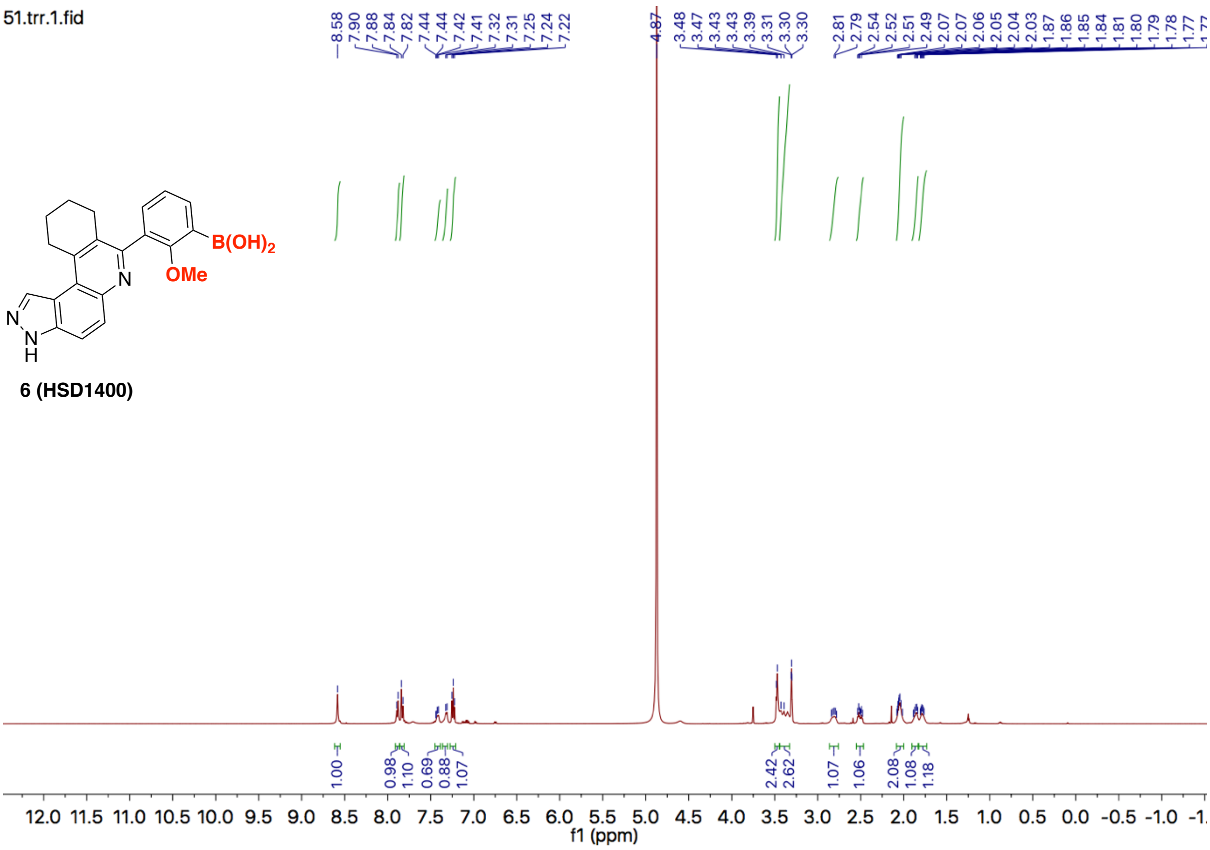

6 (HSD1400)

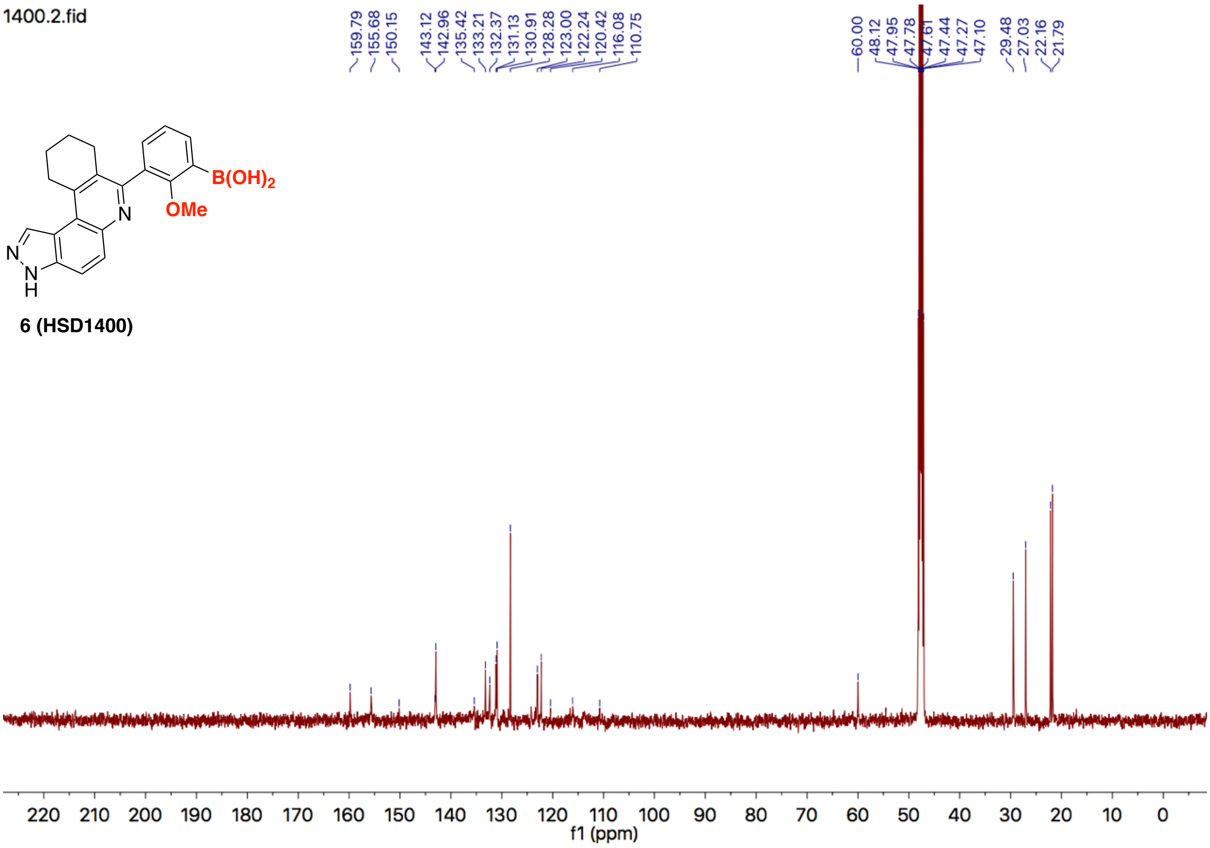
1400.2.fid
159.79
155.68
150.15
143.12
142.96
135.42
133.21
132.37
131.13
130.91
128.28
123.00
122.24
120.42
116.08
110.75
60.00
48.12
47.95
47.78
47.61
47.44
47.27
47.10
29.48
27.03
22.16
21.79
B(OH)2
OMe
N
N
H
220 210 200 190 180 170 160 150 140 130 120 110 100 90 80 70 60 50 40 30 20 10 0
f1 (ppm)

6 (HSD1400)

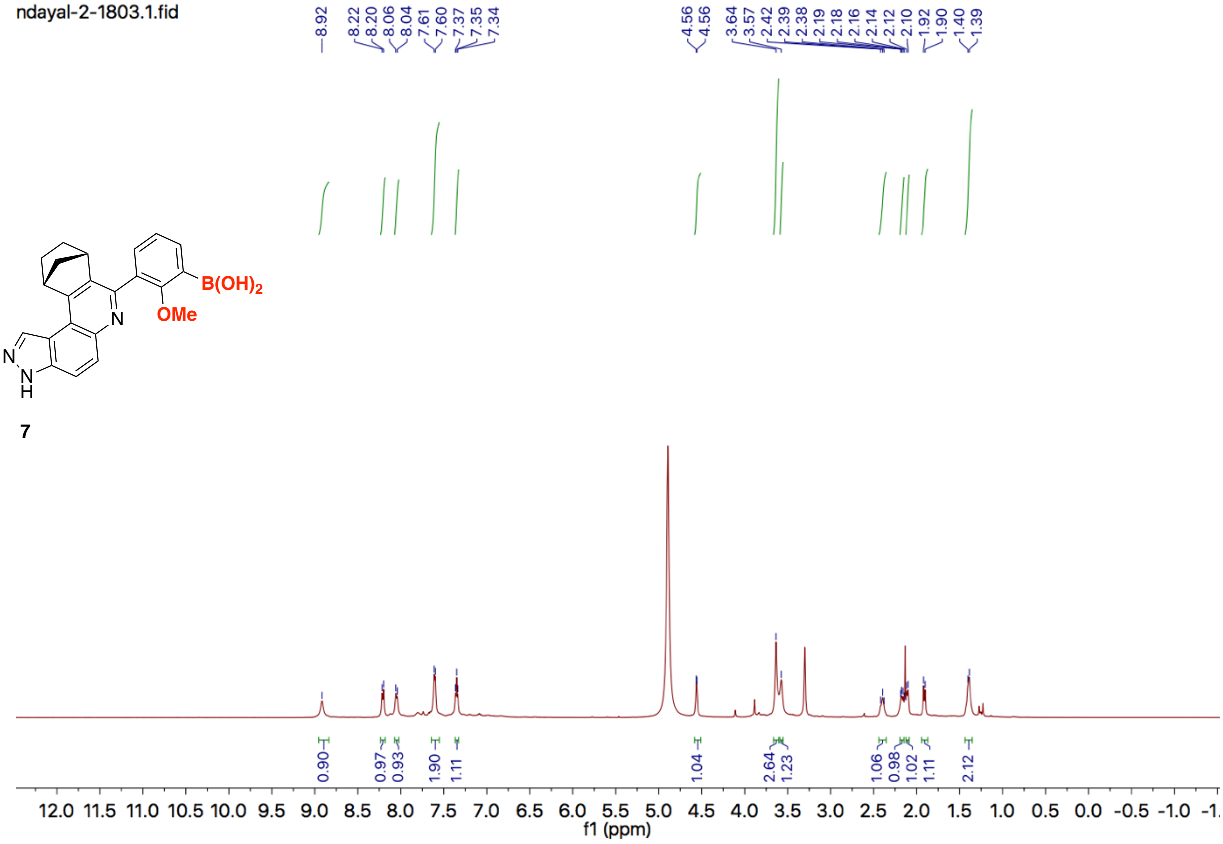
ndayal-2-1803.1.fid
8.92
8.22
8.20
8.06
8.04
7.61
7.60
7.37
7.35
7.34
4.56
4.56
3.64
3.57
2.42
2.39
2.38
2.19
2.18
2.16
2.14
2.12
2.10
1.92
1.90
1.40
1.39
B(OH)2
OMe
N
N
H
0.90
0.97
0.93
1.90
1.11
1.04
2.64
1.23
1.06
0.98
1.02
1.11
2.12
12.0 11.5 11.0 10.5 10.0 9.5 9.0 8.5 8.0 7.5 7.0 6.5 6.0 5.5 5.0 4.5 4.0 3.5 3.0 2.5 2.0 1.5 1.0 0.5 0.0 -0.5 -1.0 -1.
f1 (ppm)

7

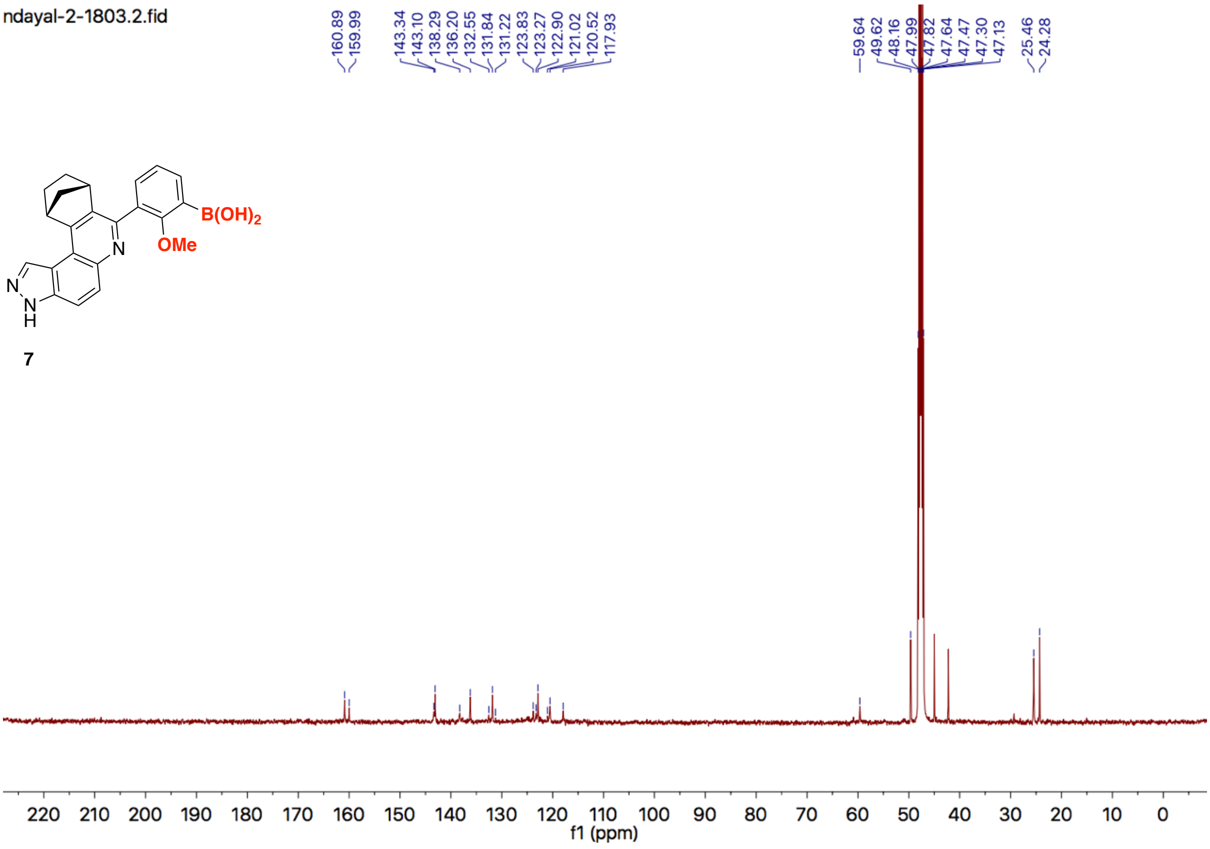
ndayal-2-1803.2.fid
160.89
159.99
143.34
143.10
138.29
136.20
132.55
131.84
131.22
123.83
123.27
122.90
121.02
120.52
117.93
59.64
49.62
48.16
47.99
47.82
47.64
47.47
47.30
47.13
25.46
24.28
B(OH)2
OMe
N
N
H
220 210 200 190 180 170 160 150 140 130 120 110 100 90 80 70 60 50 40 30 20 10 0
f1 (ppm)

7

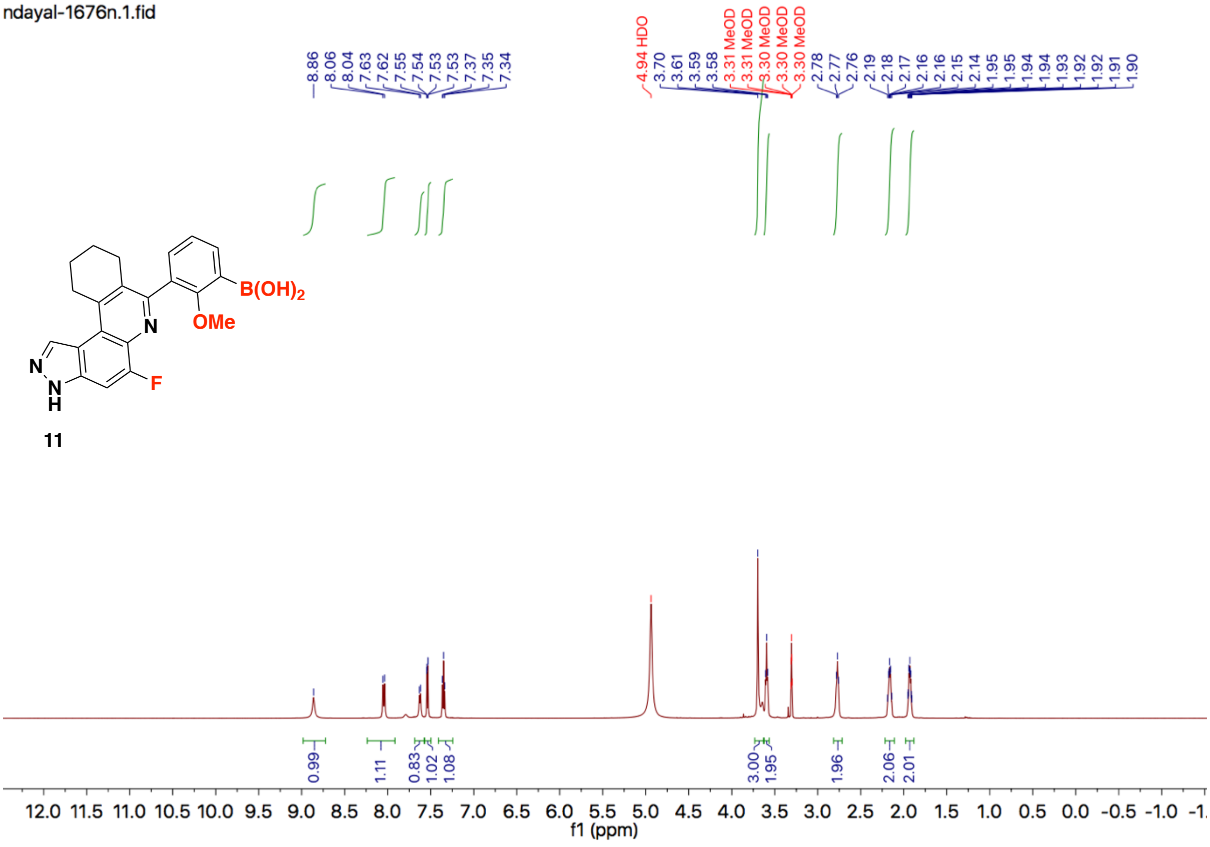
ndayal-1676n.1.fid
8.86
8.06
8.04
7.63
7.62
7.55
7.54
7.53
7.37
7.35
7.34
4.94 HDO
3.70
3.61
3.59
3.58
3.31 MeOD
3.31 MeOD
3.30 MeOD
3.30 MeOD
3.30 MeOD
2.78
2.77
2.76
2.19
2.18
2.17
2.16
2.15
2.14
1.95
1.94
1.94
1.93
1.92
1.92
1.91
1.90
B(OH)2
OMe
N
N
H
F
0.99
1.11
0.83
1.02
1.08
3.00
1.95
1.96
2.06
2.01
12.0 11.5 11.0 10.5 10.0 9.5 9.0 8.5 8.0 7.5 7.0 6.5 6.0 5.5 5.0 4.5 4.0 3.5 3.0 2.5 2.0 1.5 1.0 0.5 0.0 -0.5 -1.0
f1 (ppm)

11

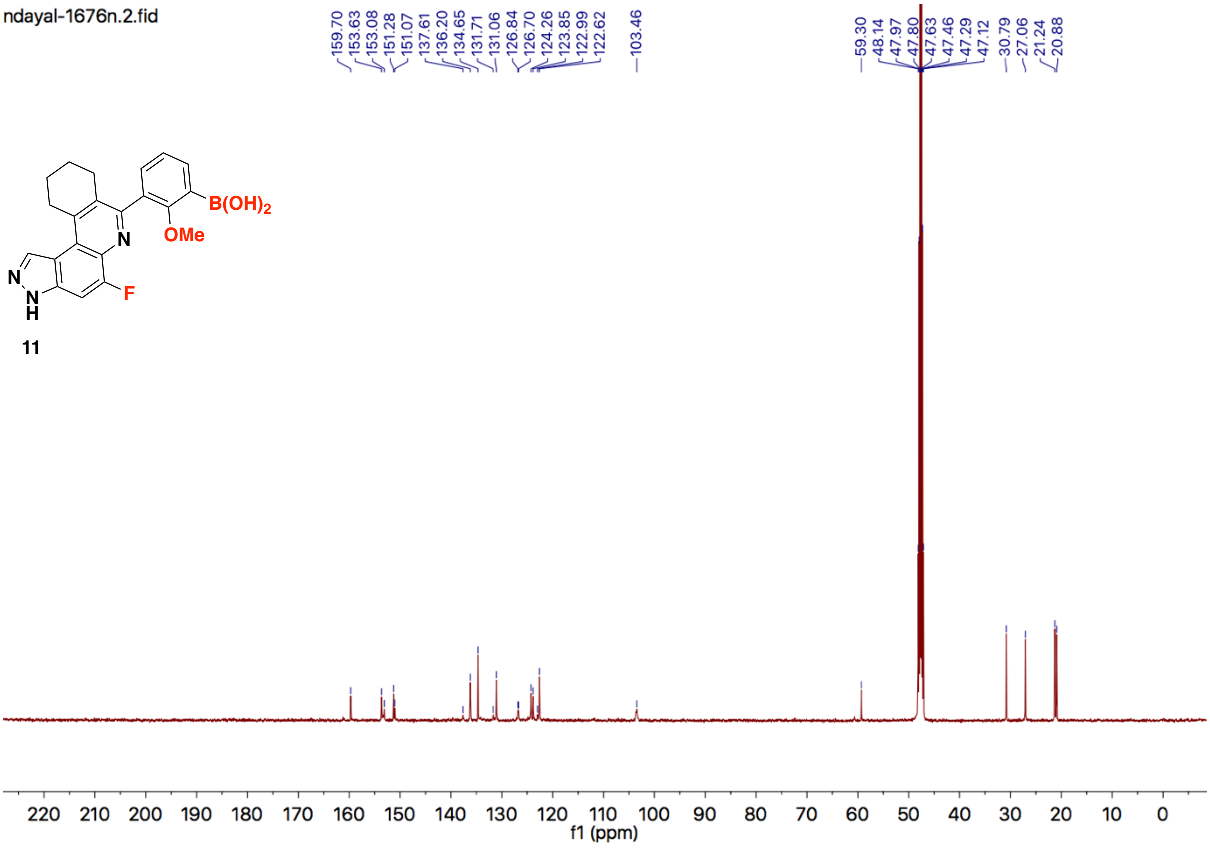
ndayal-1676n.2.fid
159.70
153.63
153.08
151.28
151.07
137.61
136.20
134.65
131.71
131.06
126.84
126.70
124.26
123.85
122.99
122.62
103.46
59.30
48.14
47.97
47.80
47.63
47.46
47.29
47.12
30.79
27.06
21.24
20.88
B(OH)2
OMe
N
N
N
H
F
11
220 210 200 190 180 170 160 150 140 130 120 110 100 90 80 70 60 50 40 30 20 10 0
f1 (ppm)

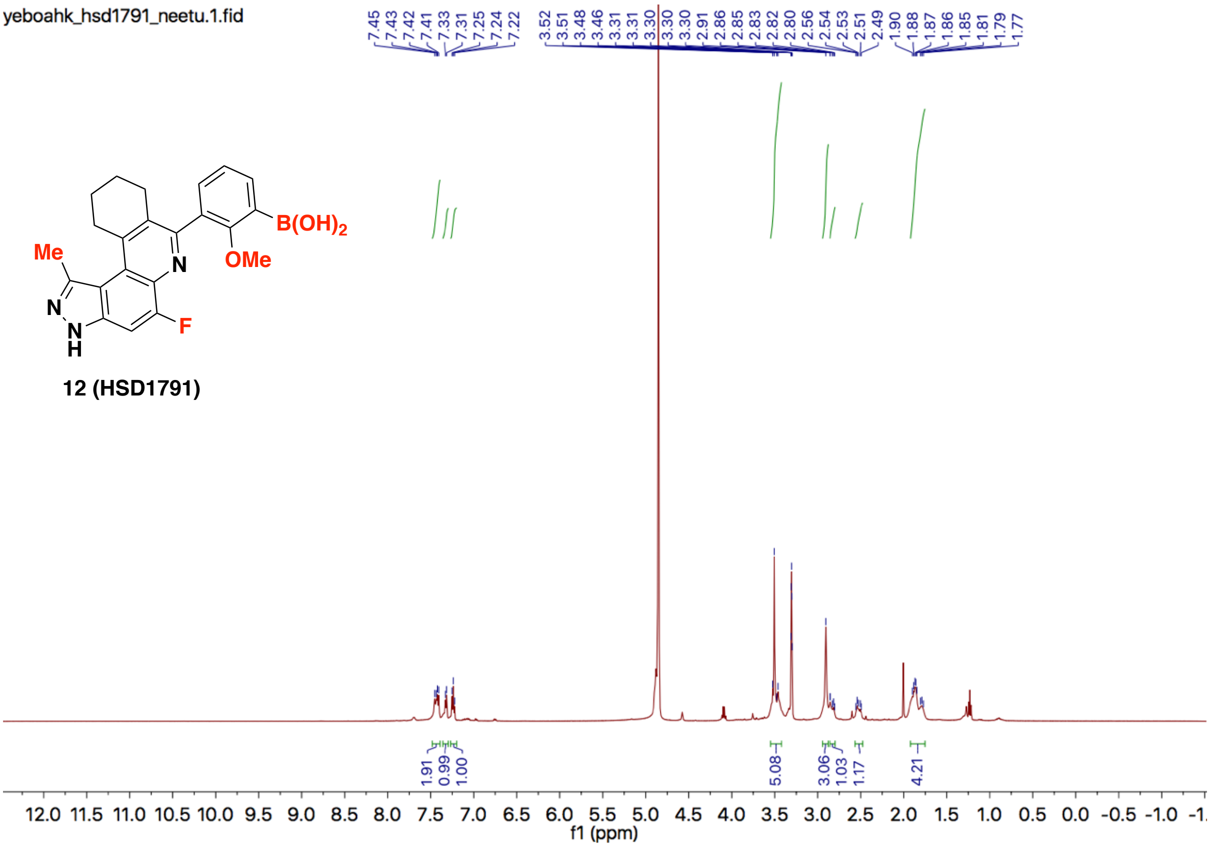
yeboahk_hsd1791_neetu.1.fid
7.45
7.43
7.42
7.41
7.33
7.31
7.25
7.24
7.22
3.52
3.51
3.48
3.46
3.31
3.31
3.30
3.30
3.30
2.91
2.86
2.85
2.83
2.82
2.80
2.56
2.54
2.53
2.51
2.49
1.90
1.88
1.87
1.86
1.85
1.81
1.79
1.77
Me
B(OH)2
OMe
N
N
N
H
F
12 (HSD1791)
1.91
0.99
1.00
5.08
3.06
1.03
1.17
4.21
12.0 11.5 11.0 10.5 10.0 9.5 9.0 8.5 8.0 7.5 7.0 6.5 6.0 5.5 5.0 4.5 4.0 3.5 3.0 2.5 2.0 1.5 1.0 0.5 0.0 -0.5 -1.0
f1 (ppm)

yeboahk_hsd1791_neetu.2.fid

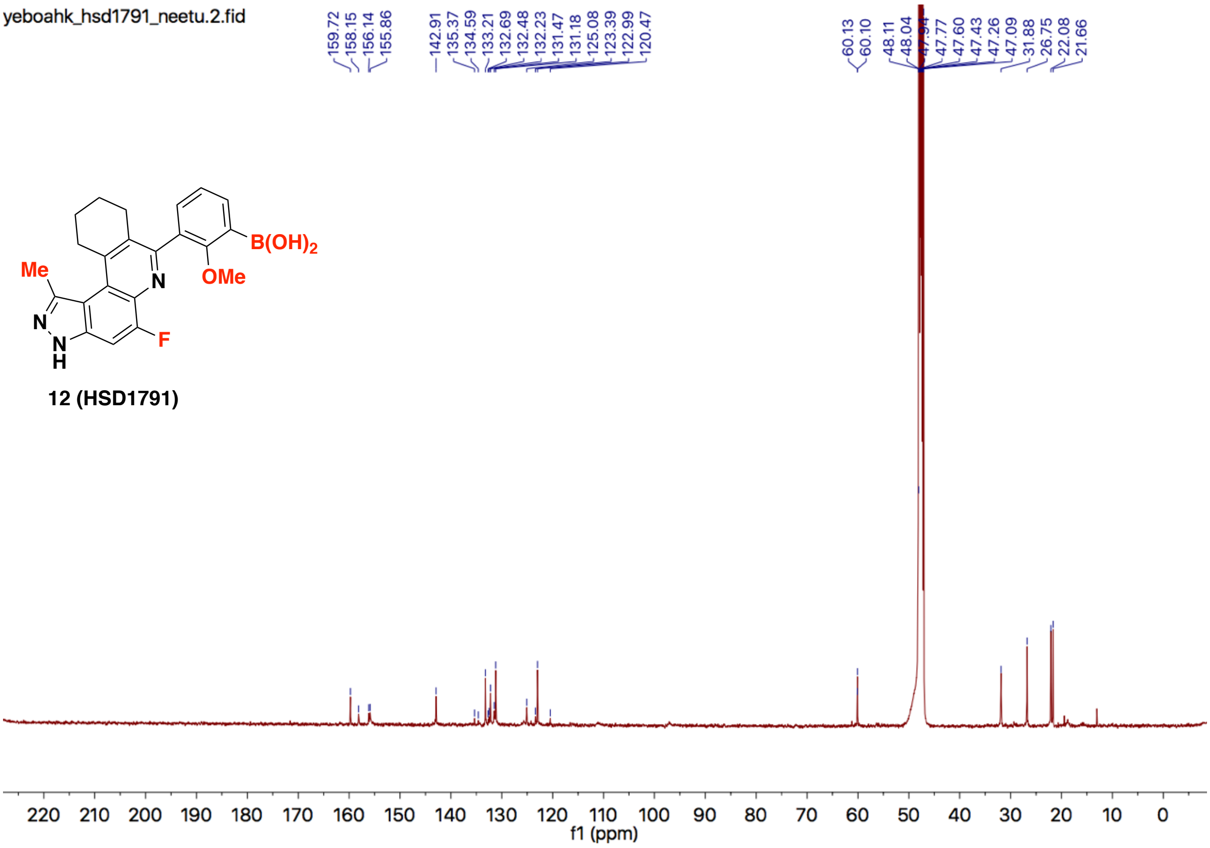

12 (HSD1791)

ndayal-4055n.1.fid

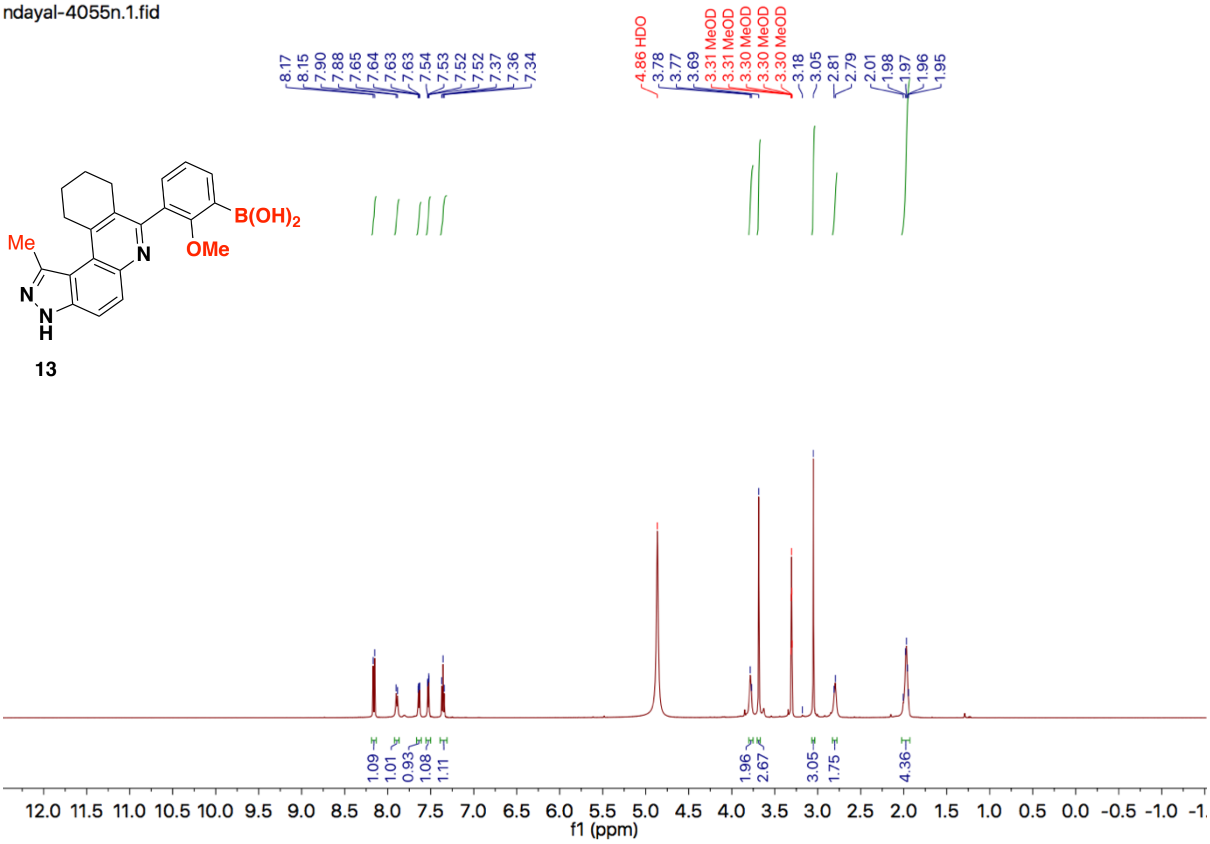

13

ndayal-4055n.2.fid

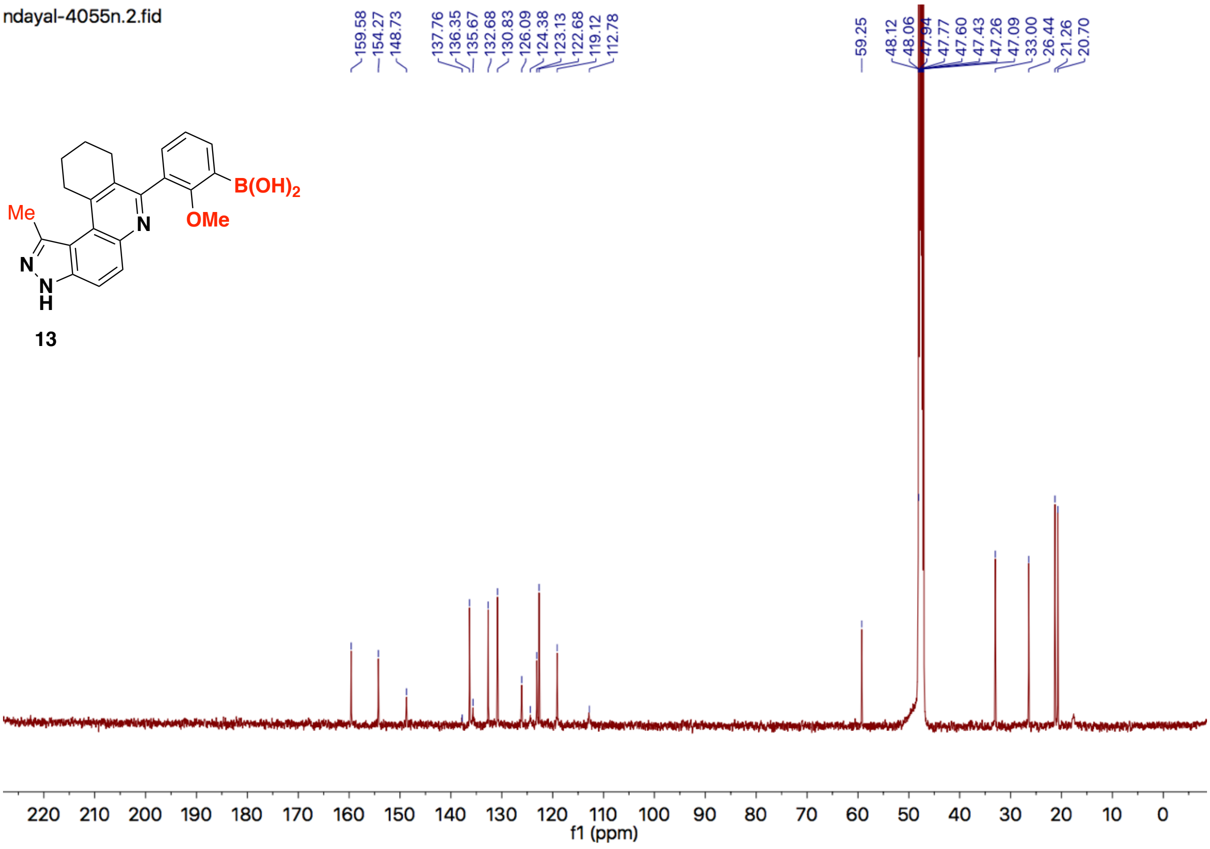

13

ndayal-1727n.1.fid

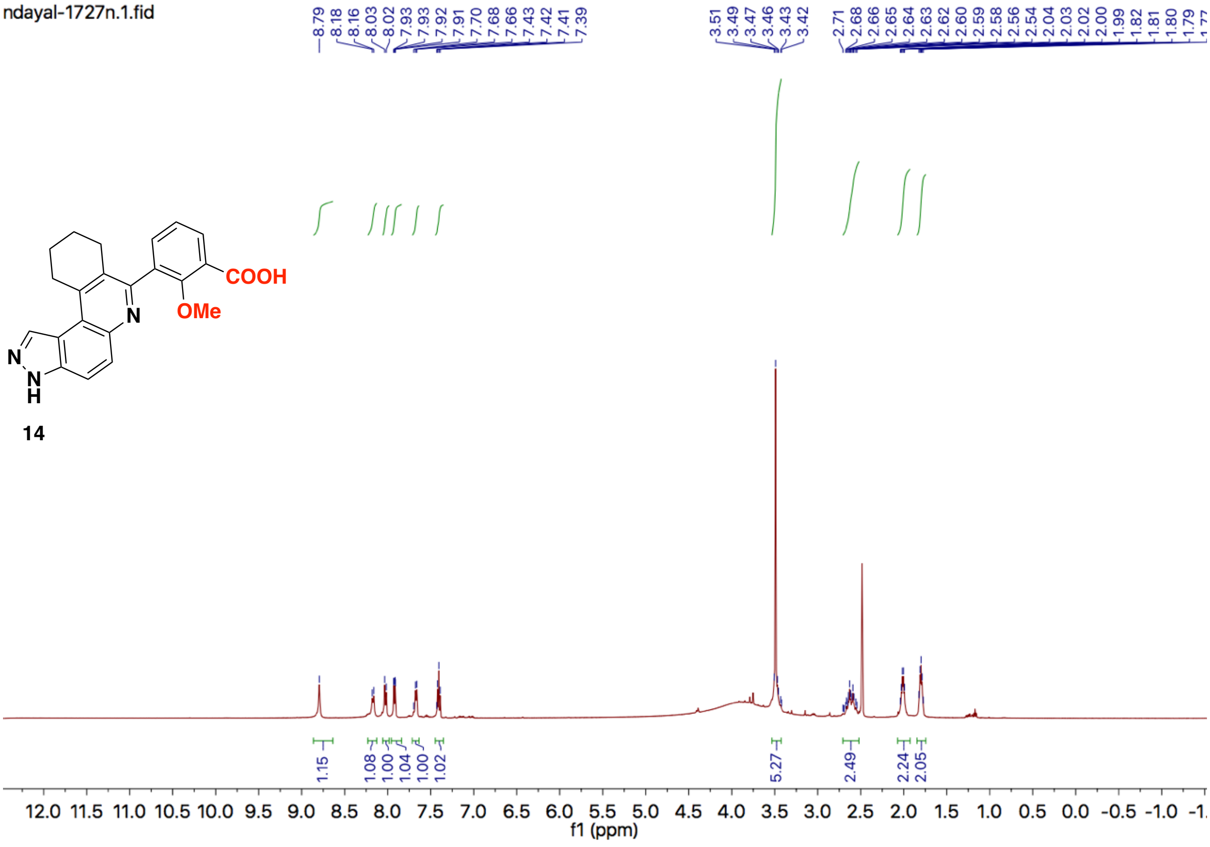

14

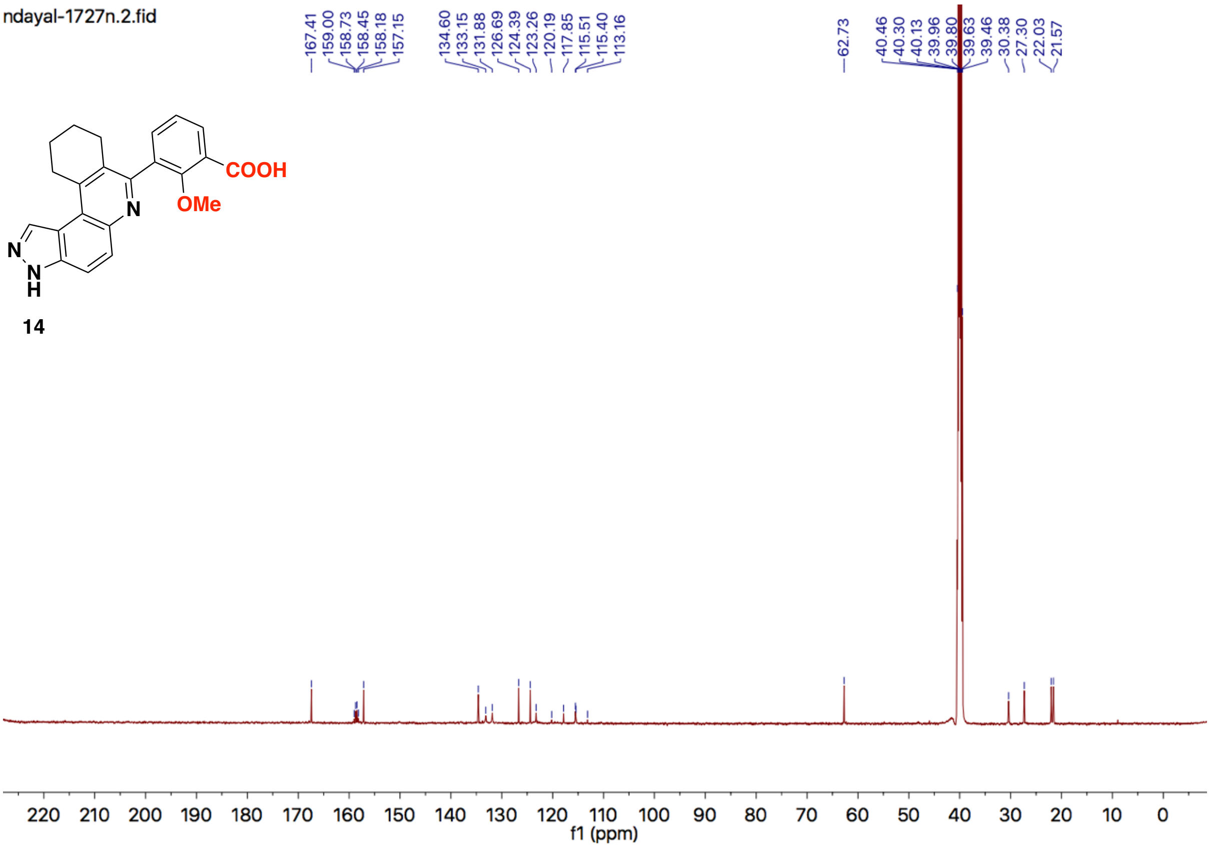
ndayal-1727n.2.fid
167.41
159.00
158.73
158.45
158.18
157.15
134.60
133.15
131.88
126.69
124.39
123.26
120.19
117.85
115.51
115.40
113.16
62.73
40.46
40.30
40.13
39.96
39.80
39.63
39.46
30.38
27.30
22.03
21.57
COOH
OMe
N
N
N
H
14
220 210 200 190 180 170 160 150 140 130 120 110 100 90 80 70 60 50 40 30 20 10 0
f1 (ppm)

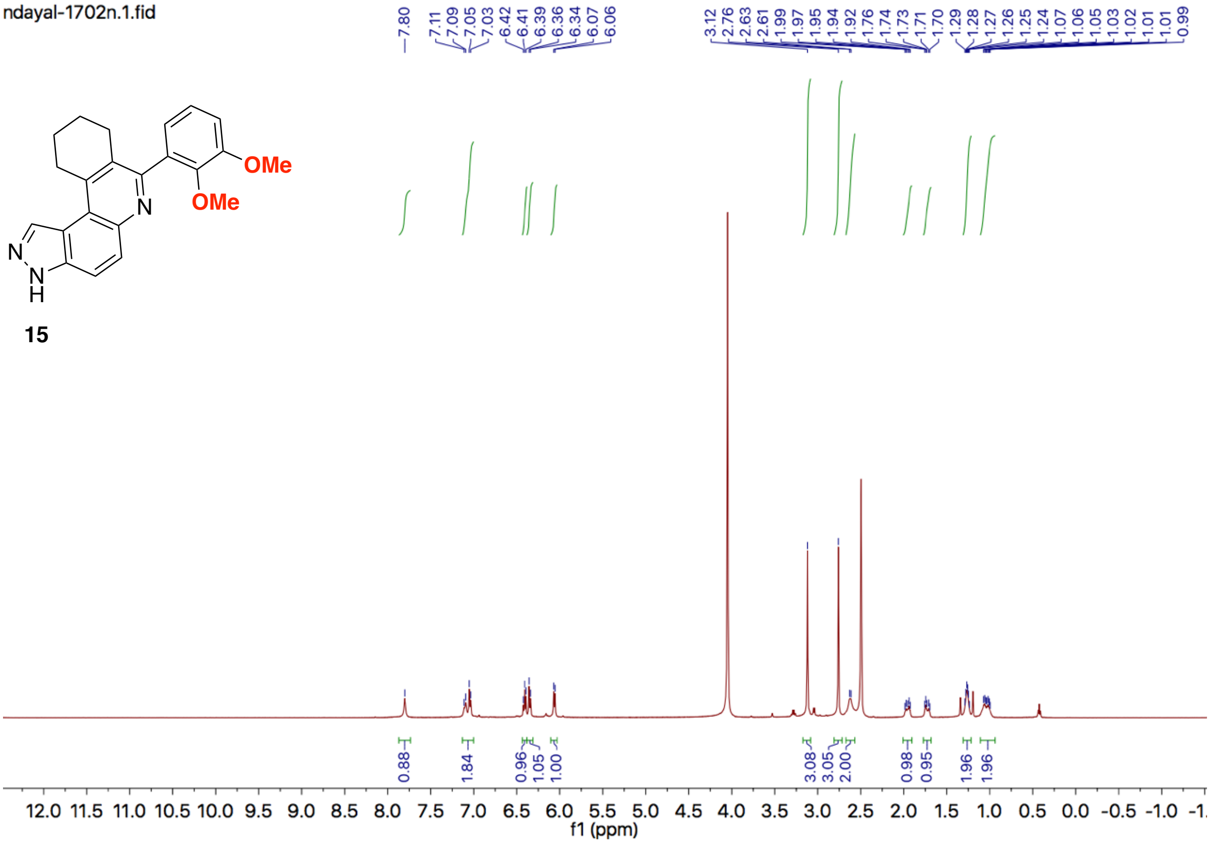
ndayal-1702n.1.fid
7.80
7.11
7.09
7.05
7.03
6.42
6.41
6.39
6.36
6.34
6.07
6.06
3.12
2.76
2.63
2.61
1.99
1.97
1.95
1.94
1.92
1.76
1.74
1.73
1.71
1.70
1.29
1.28
1.27
1.26
1.25
1.24
1.07
1.06
1.05
1.03
1.02
1.01
1.01
0.99
OMe
OMe
N
N
N
H
15
0.88
1.84
0.96
1.05
1.00
3.08
3.05
2.00
0.98
0.95
1.96
1.96
12.0 11.5 11.0 10.5 10.0 9.5 9.0 8.5 8.0 7.5 7.0 6.5 6.0 5.5 5.0 4.5 4.0 3.5 3.0 2.5 2.0 1.5 1.0 0.5 0.0 -0.5 -1.0
f1 (ppm)

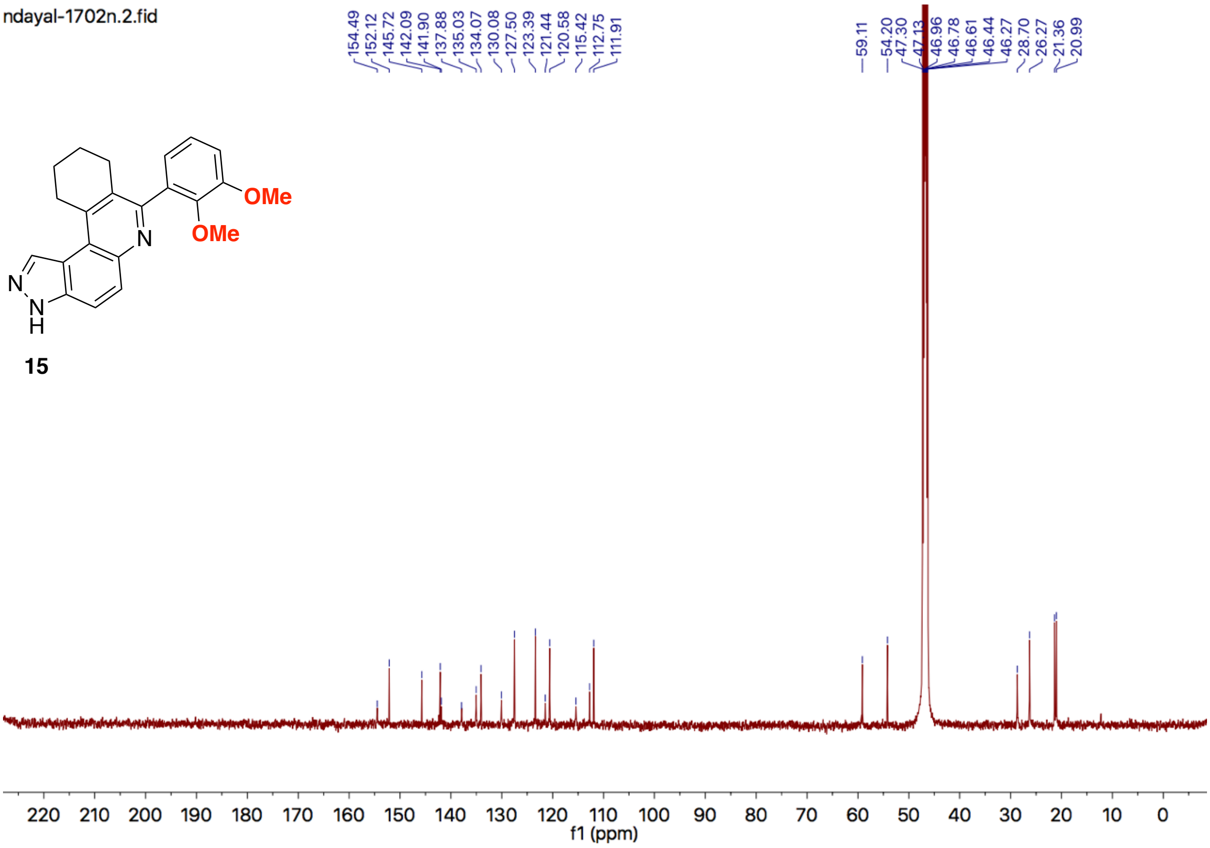
ndayal-1702n.2.fid
154.49
152.12
145.72
142.09
141.90
137.88
135.03
134.07
130.08
127.50
123.39
121.44
120.58
115.42
112.75
111.91
59.11
54.20
47.30
47.13
46.96
46.78
46.61
46.44
46.27
28.70
26.27
21.36
20.99
OMe
OMe
N
N
N
H
15
220 210 200 190 180 170 160 150 140 130 120 110 100 90 80 70 60 50 40 30 20 10 0
f1 (ppm)

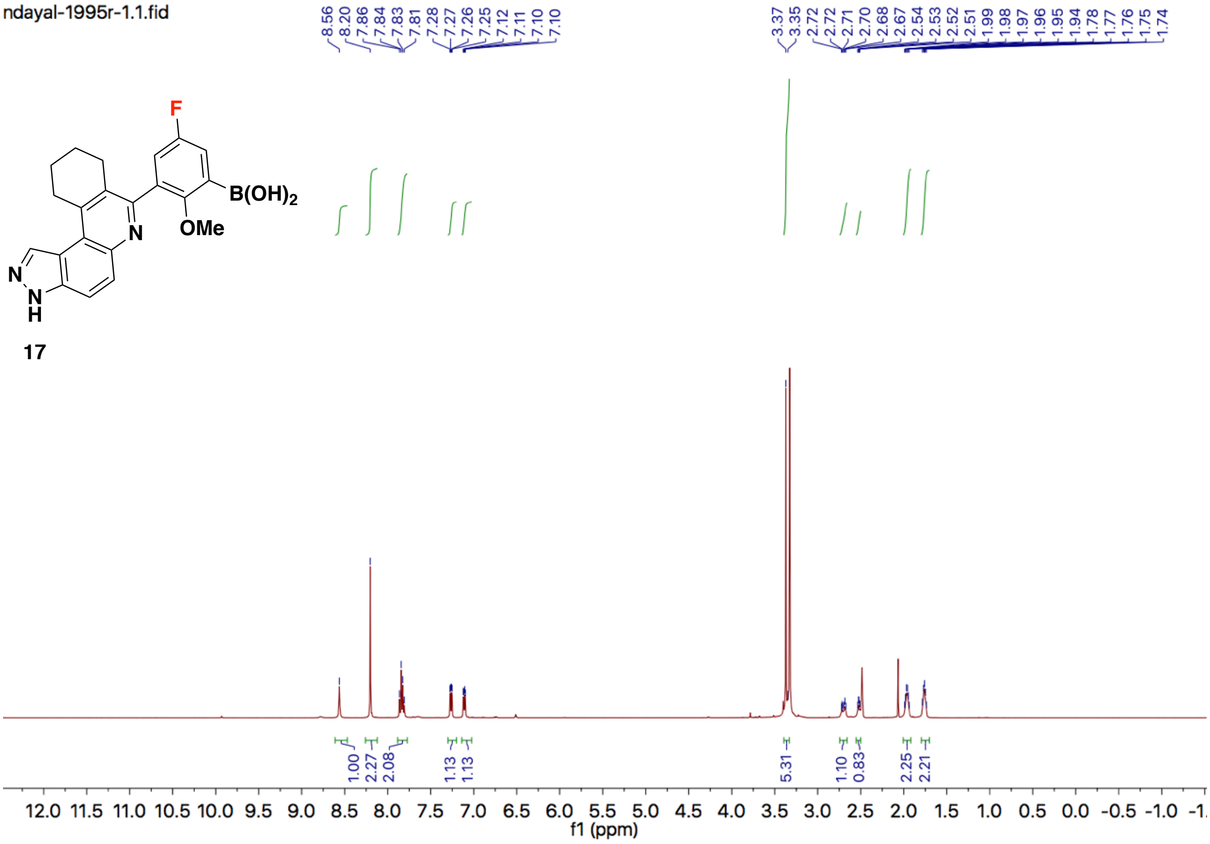
ndayal-1995r-1.1.fid
8.56
8.20
7.86
7.84
7.83
7.81
7.28
7.27
7.26
7.25
7.12
7.11
7.10
7.10
3.37
3.35
2.72
2.72
2.71
2.70
2.68
2.67
2.54
2.53
2.52
2.51
1.99
1.98
1.97
1.96
1.95
1.94
1.78
1.77
1.76
1.75
1.74
F
B(OH)2
OMe
N
N
N
H
17
1.00
2.27
2.08
1.13
1.13
5.31
1.10
0.83
2.25
2.21
12.0 11.5 11.0 10.5 10.0 9.5 9.0 8.5 8.0 7.5 7.0 6.5 6.0 5.5 5.0 4.5 4.0 3.5 3.0 2.5 2.0 1.5 1.0 0.5 0.0 -0.5 -1.0
f1 (ppm)

ndayal-1995r-1.2.fid

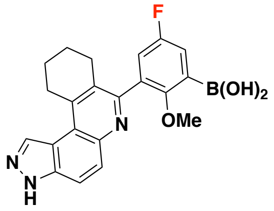

17

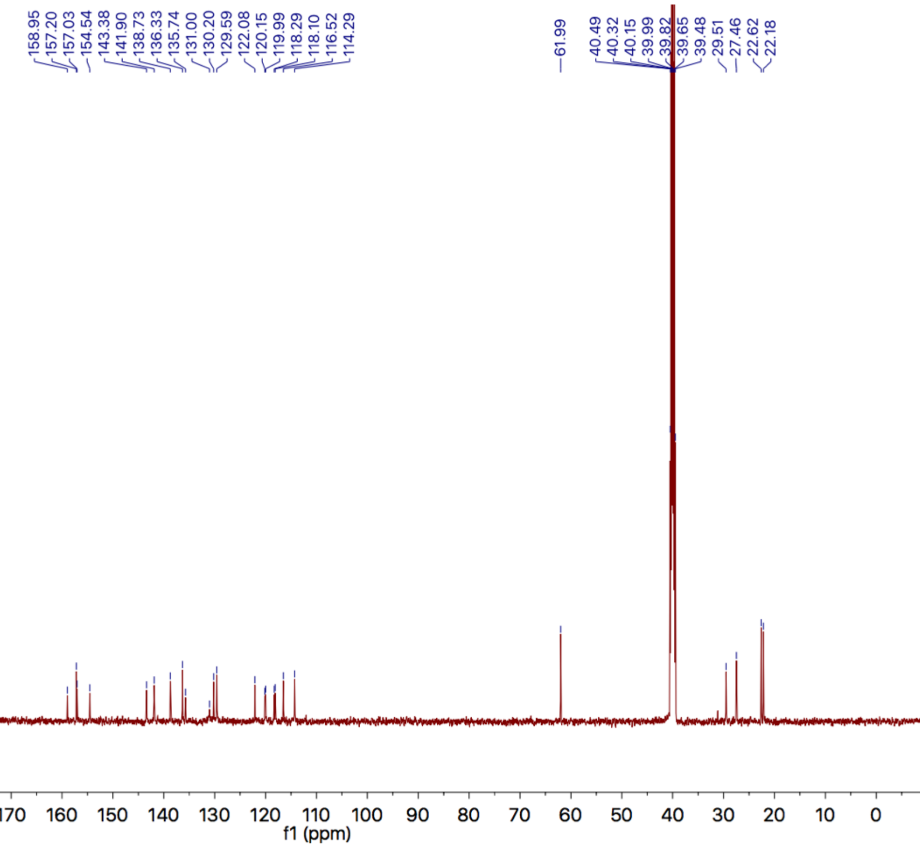

ndayal-1994n.1.fid

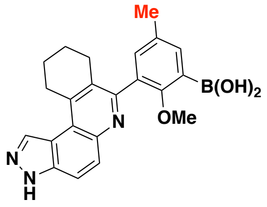

18

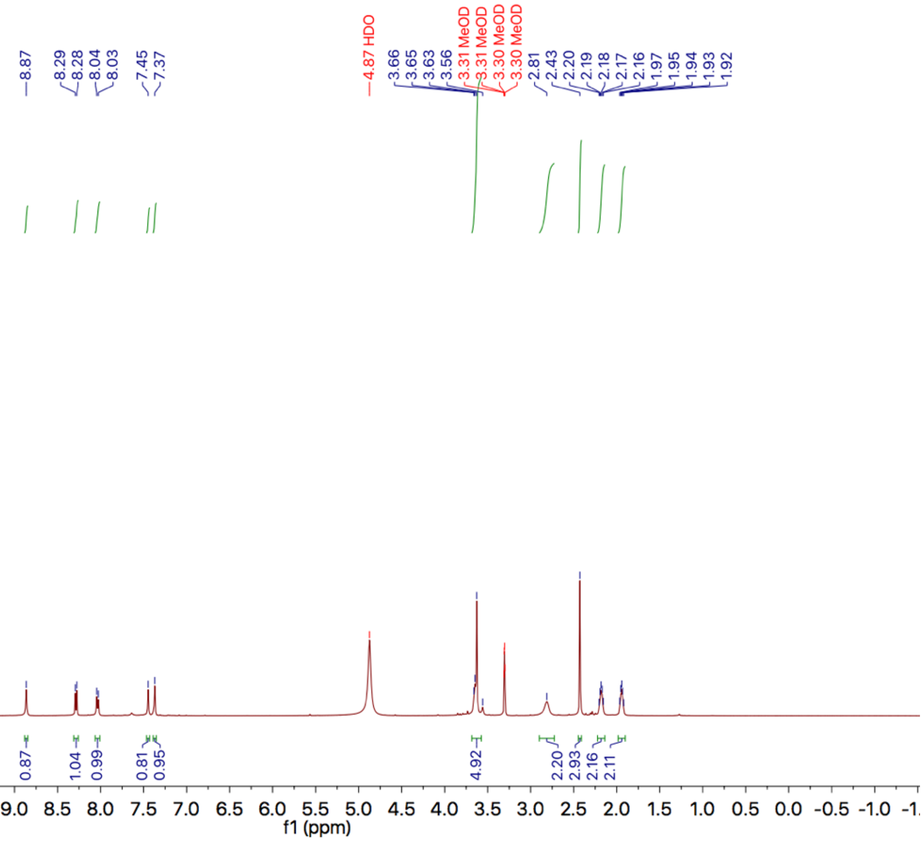

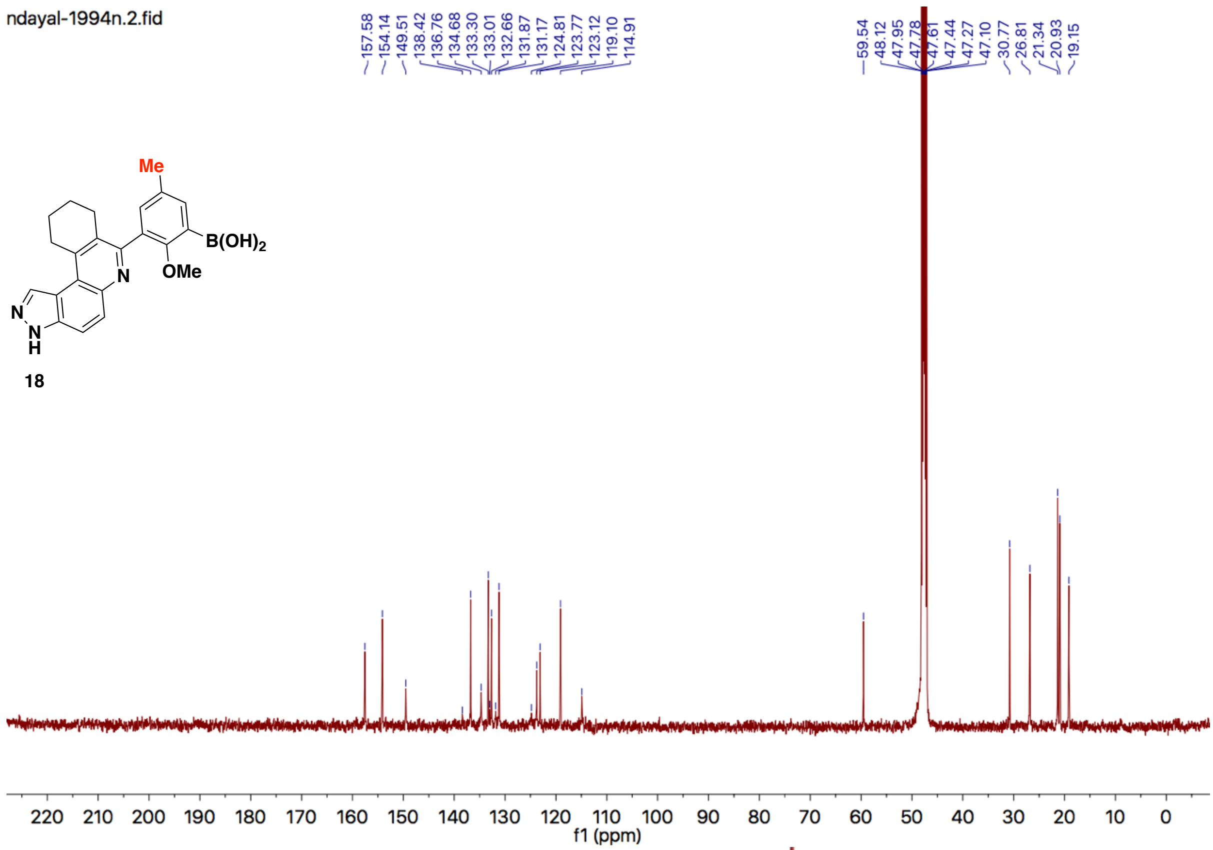
ndayal-1994n.2.fid
157.58
154.14
149.51
138.42
136.76
134.68
133.30
133.01
132.66
131.87
131.17
124.81
123.77
123.12
119.10
114.91
59.54
48.12
47.95
47.78
47.61
47.44
47.27
47.10
30.77
26.81
21.34
20.93
19.15
Me
B(OH)2
OMe
N
N
H
18
220 210 200 190 180 170 160 150 140 130 120 110 100 90 80 70 60 50 40 30 20 10 0
f1 (ppm)

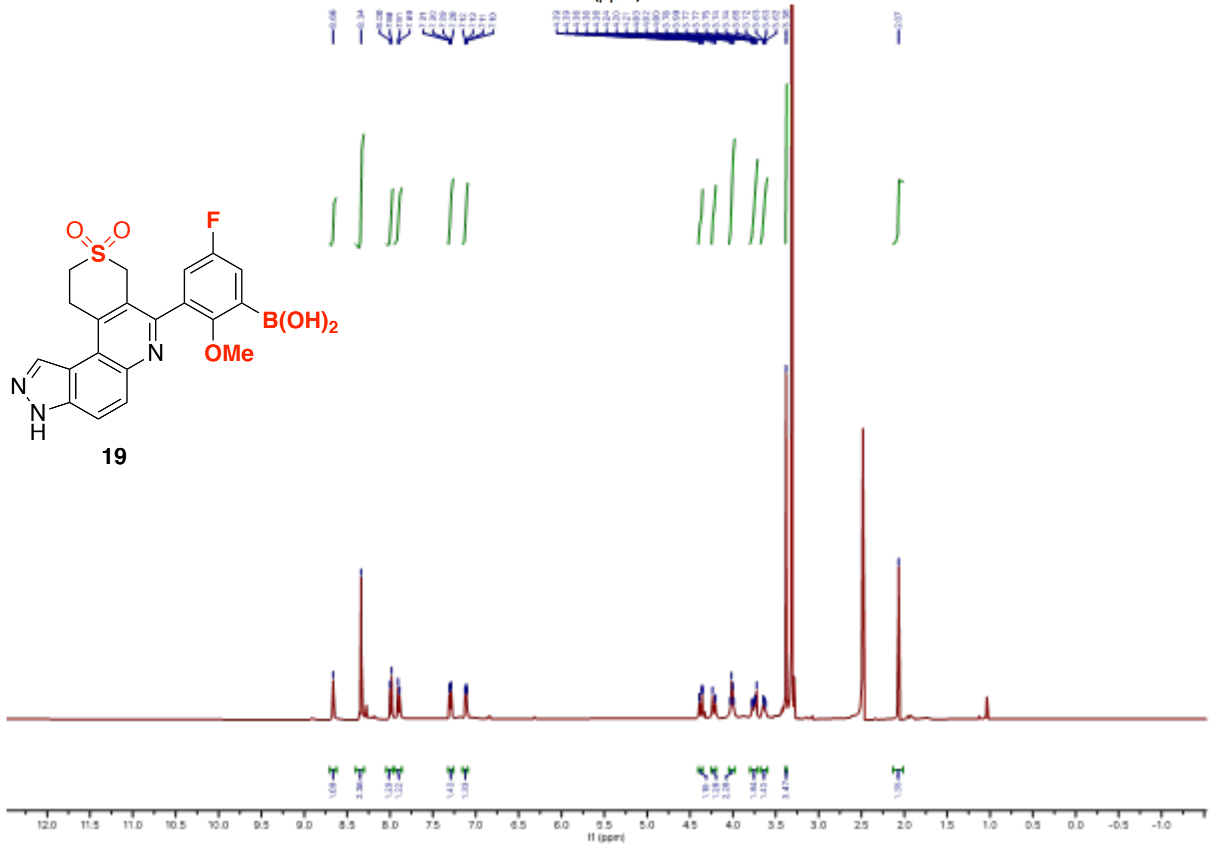
O
S
O
F
B(OH)2
OMe
N
N
H
19
12.0 11.5 11.0 10.5 10.0 9.5 9.0 8.5 8.0 7.5 7.0 6.5 6.0 5.5 5.0 4.5 4.0 3.5 3.0 2.5 2.0 1.5 1.0 0.5 0.0 -0.5 -1.0
f1 (ppm)

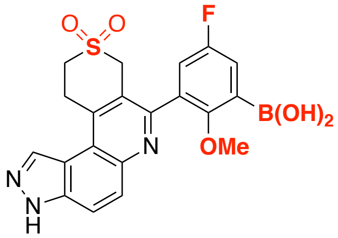

19

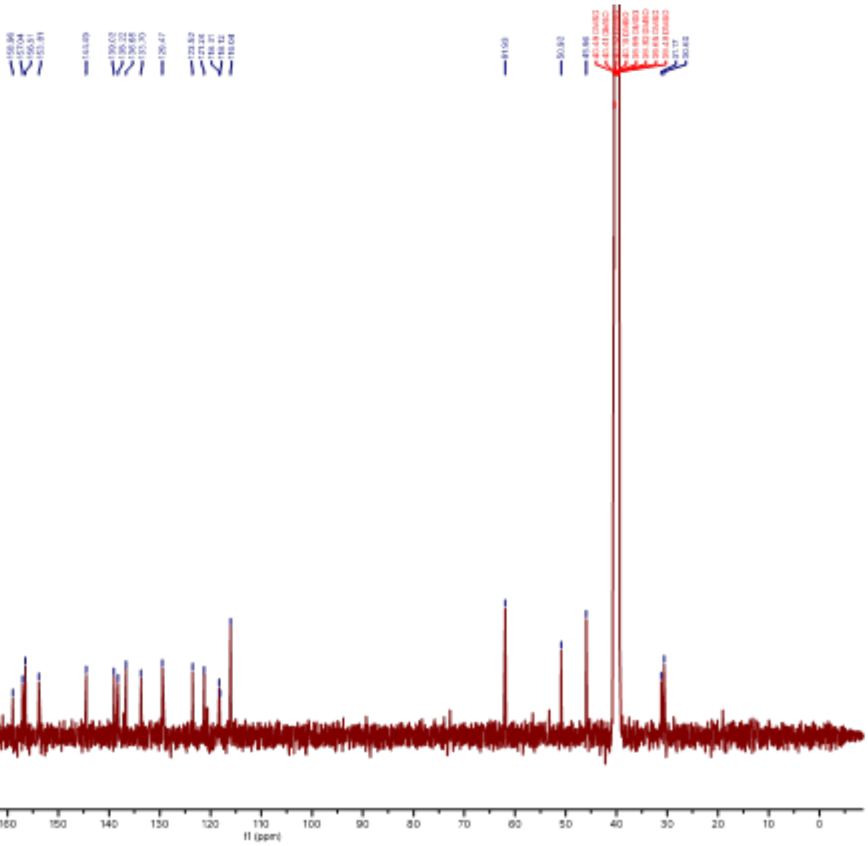

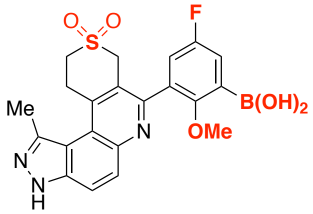

20

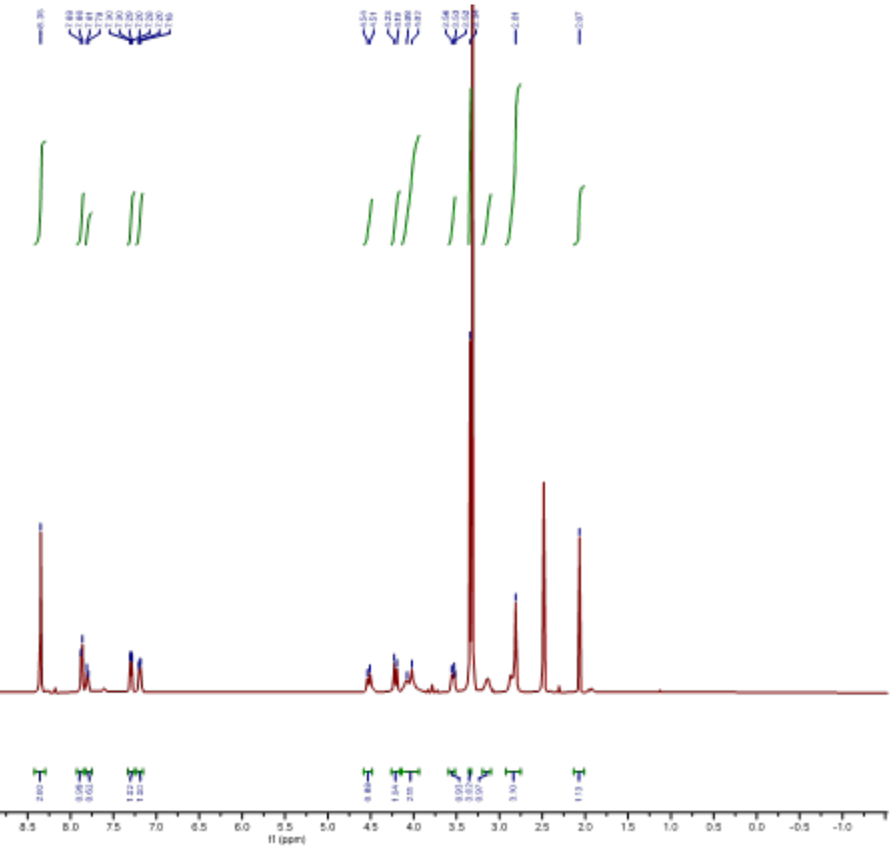

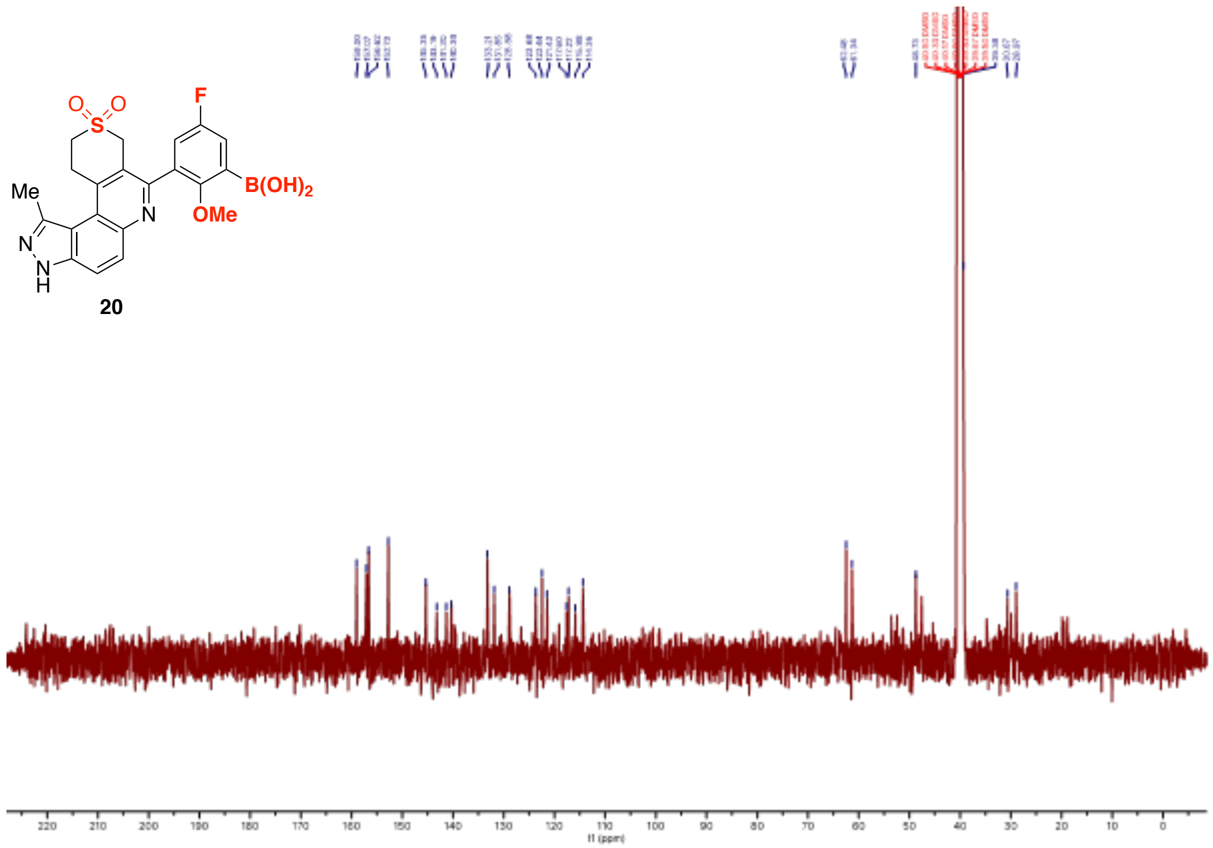

Supplement: Supplementary file 1 [file pharmaceuticals-17-01660-s001.zip › pharmaceuticals-3305023-supplementary.pdf]
